# Digital empowerment, subsidy coordination, and food security: a tripartite evolutionary game with simulation analysis

**DOI:** 10.3389/fnut.2026.1887202

**Published:** 2026-07-29

**Authors:** Qian Qu, Yuntao Tan, Liping He, Hong Wang, Deliang Tang, Jixin Fan

**Affiliations:** 1 Chongqing Industry Polytechnic University, Chongqing, China; 2 School of Business Administration, Chongqing Technology and Business University, Chongqing, China; 3 Department of Electronic Audio-Visual Media, Chongqing University Press, Chongqing, China

**Keywords:** digital technology, empowerment effect, food security, government subsidies, three-player evolutionary game

## Abstract

Against the backdrop of the deep integration of digital technology and agriculture and the coordinated advancement of the national food security strategy, the effectiveness of digital technology in enhancing food security increasingly hinges on the interaction and coordination among local governments, digital agriculture service providers, and grain producers. This paper constructs a three-party evolutionary game model that incorporates key variables, including the digital empowerment coefficient, the technology accessibility coefficient, differentiated subsidy parameters, and central government incentives. It systematically analyzes the evolutionary trajectories of the three parties' strategies and the conditions for steady-state transitions under various parameter combinations. Furthermore, it conducts numerical simulations using benchmark parameters calibrated through multi-source data fusion. The research reveals four typical evolutionary scenarios. First, when the technology is effective and the service is profitable, the system converges toward an ideal steady state of tripartite collaboration. Second, when the technology is effective but the service is less profitable, the steady state hinges on whether subsidies can offset cost disadvantages and on the strength of government incentives. Third, when there is sufficient momentum on the service side, but the technology lacks appeal, the core bottleneck lies in stimulating key stakeholders' willingness to adopt it. Finally, when both technological effectiveness and market profitability are weak, the system risks becoming locked into a low-level equilibrium. Benchmark parameter calculations indicate that China's major grain-producing regions currently exhibit the characteristic of being “technologically effective but with weak profitability among service providers,” and that the system possesses an intrinsic drive to converge toward an ideal steady state. Sensitivity analysis reveals four key levers driving the system's evolution: the digital empowerment coefficient, the intensity of subsidies targeting primary actors (grain producers), service providers' operating costs, and the scale of central government incentives. Therefore, policy design should shift from a single-instrument reliance to a differentiated and coordinated approach, tailoring implementation strategies to the specific stage of regional development. A portfolio of policy instruments, centered on these key parameters, should be developed to integrate demand-side incentives, supply-side support, and government performance assessments. Furthermore, facilitating the cross-regional transfer of theoretical models and policy experience can provide a sustainable driver for leveraging digital technologies to enhance food security.

## 1 Introduction

Food security is a fundamental pillar of national security, an assessment that applies not only to China but also to the majority of developing countries worldwide. Over the past decade, China has sustained high and stable levels of grain production by implementing the strategy of “storing grain in the land and in technology” (i.e., building production capacity through farmland improvement and technological advancement). However, tightening resource and environmental constraints, an accelerating aging of the agricultural workforce, and growing uncertainties associated with climate change have collectively pushed the traditional input-based production model ever closer to its limits. Amid the various efforts to address these challenges, digital technologies—such as big data, artificial intelligence, and the Internet of Things—are rapidly penetrating the agricultural sector. They offer new technical solutions to alleviate efficiency bottlenecks, tackle risk management challenges, and improve resource allocation inefficiencies in food production. From an international perspective, digital agriculture is also seen as a key pathway to mitigating the effects of climate change and increasing food self-sufficiency. In their review, Balasundram et al. (1) note that digital agricultural technologies can increase output per unit area while reducing greenhouse gas emissions, thereby offering a technical pathway to balancing food security with environmental sustainability. However, the actual uptake of these technologies varies significantly across countries at different stages of development, with weak infrastructure and inadequate institutional frameworks often posing key constraints. A review of low-and middle-income countries by Manzoor et al. (2) further reveals that agro-ecological conditions, institutional environments, and socio-cognitive and behavioral factors collectively shape the effectiveness of digital technology adoption. The mere provision of technology is unlikely to translate into increased food production.

In response to the dual challenges of climate change and food insecurity, both international organizations and national governments have intensified their policy efforts to harness digital technologies for agricultural transformation. At the global level, the Food and Agriculture Organization released The Impact of Disasters on Agriculture and Food Security 2025, which demonstrates how satellite monitoring, artificial intelligence, mobile advisory services, and predictive analytics are revolutionizing agricultural disaster risk management and shifting the paradigm from reactive response to proactive prevention. The European Commission presented its “Vision for Agriculture and Food” in February 2025, an ambitious roadmap that includes a forthcoming EU digital strategy for agriculture to support the transition to digital-ready farming. The G20 Agriculture Working Group and Food Security Task Force, under South Africa's 2025 presidency, adopted the Ubuntu Declaration, reaffirming commitment to multilateralism and the 2030 Sustainable Development Goals, with technology and innovation featuring prominently, including calls for stronger digital systems to improve market transparency and early warning mechanisms. At the national level, China's 2025 Central Document No. 1 proposed “developing agricultural new quality productive forces” and supporting the development of smart agriculture by expanding the application scenarios for artificial intelligence, data, and low-altitude technologies. In May 2025, the Office of the Central Cyberspace Affairs Commission, together with the Ministry of Agriculture and Rural Affairs, the National Development and Reform Commission, and the Ministry of Industry and Information Technology, jointly issued the 2025 Key Tasks for Digital Rural Development, which sets the goal of further manifesting the role of digital technologies in ensuring national food security, with 26 key tasks across nine areas. These policy developments underscore the growing recognition that digital technologies offer a critical pathway to enhancing food security; however, their effectiveness depends on coordinated actions by multiple stakeholders, which calls for systematic analysis from a game-theoretic perspective.

Digital technology plays a vital role in enhancing food security across multiple stages, including production, management, and marketing. On the production side, technologies such as precision fertilization, smart irrigation, and drone-based crop protection have been shown to improve resource-use efficiency and yield per unit area significantly (3, 4). On the management side, agricultural big data platforms strengthen the risk resilience of grain production systems by enabling real-time monitoring and early warning of soil moisture, crop conditions, and natural disasters. On the market side, the expansion of digital financial tools and e-commerce channels helps alleviate the financing constraints and market access difficulties faced by grain producers (5). However, a growing body of research recognizes that transforming digital technologies from potential productivity gains into tangible food security is not an automatic process of technology transfer. Rather, it is a complex social construction, deeply shaped by the interplay of diverse stakeholder interests and constrained by the institutional environment. An empirical study by Peng and Huang (6), drawing on provincial panel data from China, indicates that the impact of digital technologies on grain yield per unit area is mediated by multiple channels, including the level of socialized agricultural services, the standard of field management, and the degree of agricultural mechanization, with the scale of agricultural operations acting as a positive moderator. This implies that the actual effectiveness of digital technologies in strengthening food security depends not only on the maturity of the technologies themselves, but also on the synergistic interaction among structural factors such as service provision, supporting institutional frameworks, and the capabilities of key stakeholders. A study by Choruma et al. (7) on smallholder farmers in sub-Saharan Africa further supports this view, demonstrating that in regions with poor infrastructure and inadequate institutional safeguards, the effectiveness of digital technology adoption is often severely constrained.

A considerable body of research has examined the relationship between digital technology and food security. Some studies have examined the impact of the digital economy on agricultural total factor productivity from a macroeconomic perspective, confirming the positive contribution of digital technologies to food production capacity. Another body of research adopts a micro-level lens, focusing on individual farmers and identifying perceived usefulness, perceived ease of use, and external support conditions as key variables shaping willingness to adopt technology. However, most existing research remains confined to analyzing the behavior of individual actors or testing direct “technology-output” relationships. Few studies have systematically examined local governments, digital service providers, and grain producers within a unified game-theoretic framework. Notably, food security exhibits strong public-good characteristics: local governments' behavior is governed by both political incentives and fiscal constraints; service providers' supply decisions are closely tied to market expectations; and heterogeneous factors, such as risk perception and resource endowments shape grain producers' technology adoption. These three-pronged strategies are interdependent and subject to dynamic feedback loops, and the outcome of their evolutionary equilibrium directly determines the actual effectiveness of digital technology in strengthening food security. Regarding the application of evolutionary game theory, Sun et al. (8) constructed a model of strategic interaction between digital technology service providers and agricultural operators under government incentives, identifying the evolutionarily stable conditions for subsidy intensity and service providers' levels of effort. Zheng et al. (9) further developed a three-party evolutionary game model involving the government, digital technology firms, and farmers' cooperatives to explore the dynamic matching mechanism between supply-side service quality and demand-side willingness to adopt. The aforementioned studies have provided an important methodological foundation for applying evolutionary game theory to digital agricultural governance. Nonetheless, research that simultaneously places local governments, digital service providers, and grain producers within one unified game-theoretic framework-while specifically examining how digital technology can strengthen food security-remains relatively scarce.

While the existing literature has provided important methodological foundations, several gaps remain unaddressed. Sun et al. (8) focused on the interaction between service providers and agricultural operators under government incentives, while Zheng et al. (9) explored the matching mechanism between supply-side service quality and demand-side adoption willingness. However, these studies do not explicitly link digital technology adoption to food security outcomes, nor do they incorporate key variables such as digital empowerment effects and technology accessibility.

Based on the above analysis, this paper constructs a three-party evolutionary game model that encompasses local governments, digital agriculture service providers, and grain producers. By systematically characterizing the enabling effects of digital technology, government subsidy incentives, service providers' revenue structures, and the costs and benefits to grain producers, this paper analyzes the evolutionary trajectories of the three parties' strategies and the corresponding steady-state conditions. Specifically, it seeks to answer the following questions: under what parameter configurations can the system converge to the ideal steady state characterized by “strong government support, high-quality service provision, and active technology adoption by grain producers.” How do the effects of digital empowerment, the intensity of government subsidies, and the revenue structure of service providers interact to steer the direction of system evolution? How should policy instruments be designed to guide the behavior of these three parties toward coordination effectively? Answering these questions is of considerable theoretical value and practical significance for refining the digital agriculture policy framework and promoting the deep integration of digital technologies with food security.

## 2 Theoretical framework and model development

### 2.1 Theoretical framework

#### 2.1.1 Technology acceptance models and theories of farmer behavior

The technology acceptance model (TAM) posits that an individual's willingness to adopt a new technology depends primarily on two core variables: perceived usefulness and perceived ease of use (10–12). In agricultural technology extension, TAM is widely used to explain farmers' adoption of new technologies and crop varieties. Perceived usefulness refers to farmers' subjective assessment of whether digital technologies can effectively increase yields, reduce costs, and mitigate risks. Perceived ease of use, meanwhile, reflects farmers' evaluation of a technology's complexity, learning curve, and the accessibility of supporting services. Biondo et al. (13) integrated the TAM2 model with risk attitude in an empirical study of Sicilian winegrowers. They found that perceived usefulness and perceived ease of use remain the most significant factors influencing technology adoption, and that risk-averse tendencies significantly reduce farmers' willingness to adopt precision agriculture technologies. The theory of farmer behavior further suggests that a dual logic of profit-seeking and risk-aversion drives smallholders' production decisions. A study by Patil and Veettil (14) of rice farmers in eastern India indicates that risk-seeking farmers are more inclined to adopt capital-intensive technologies such as mechanization, while risk-averse farmers are more likely to opt for low-capital-threshold technologies, such as stress-tolerant varieties. This implies that, when promoting the adoption of digital technologies, it is essential not only to highlight the expected benefits but also to reduce farmers' perception of risk through measures such as insurance and subsidies.

#### 2.1.2 Incentive-compatibility theory

Rooted in game theory, the central tenet of incentive compatibility theory is that an institutional arrangement should enable individuals, while pursuing their own self-interest, to achieve collective or societal objectives simultaneously. In the context of digital technology-enabled food security governance, incentive compatibility entails harmonizing three objectives. First, performance appraisal mechanisms for local governments should provide an intrinsic incentive to promote digital agriculture development while pursuing regional development and fiscal sustainability. Second, the business models of digital agriculture service providers should provide a sustained incentive to deliver high-quality, wide-reaching technical services while pursuing commercial profits. Third, the income structures of grain producers should encourage the voluntary adoption of digital technologies that enhance grain production capacity while maximizing household income. Liao et al. (15) employed evolutionary game theory to construct a three-party model involving local governments, agricultural enterprises, and consumers. They found that the system can evolve toward an ideal equilibrium state of tripartite cooperation only when incentive-compatibility conditions are met, and they further identified the optimal ranges for the subsidy coefficient and carbon penalty. This finding underscores the central role of incentive-compatibility theory in agricultural governance. Specifically, the degree of incentive compatibility among the three parties directly determines the likelihood that digital technology can enable food security to transition from a “policy requirement” to an “endogenous action.” If institutional design fails to achieve incentive compatibility, the system risks becoming trapped in a low-level equilibrium.

#### 2.1.3 Stakeholder theory

Stakeholder theory posits that the effective functioning of any organization or system depends on identifying, balancing, and addressing the demands of diverse stakeholders (16). In the governance context of digital technology-enabled food security, local governments, digital agriculture service providers, and grain producers constitute three key stakeholder groups, each with distinct resource endowments, objectives, and rationales for action. Local governments, which control policy resources and fiscal instruments, primarily seek to strike a balance between political accountability for food security and the sustainability of local finances. Digital agriculture service providers that control technological resources and market channels primarily aim to achieve a positive return on investment. Grain producers, who control land and labor resources, primarily seek to make sound decisions that balance the expected income gains from adopting new technologies against production risks. Drawing on a three-party evolutionary game model, Xu (17) examined the strategic interactions among the government, digital technology providers, and rural industrial actors in the digital transformation of rural industries. The study found that the evolutionary steady state of the three-party game ultimately converges to an ideal equilibrium characterized by government incentives, proactive supply from technology providers, and active adoption by rural industrial actors. This study provides direct theoretical support for analyzing multi-stakeholder strategic interactions and policy design from a stakeholder perspective.

#### 2.1.4 Synthesizing the three theoretical pillars

The three theoretical pillars introduced above jointly inform our modeling framework rather than standing alone. The Technology Acceptance Model guides the specification of producer behavior by linking perceived usefulness to the expected yield gain and success probability under digital adoption, and perceived ease of use to accessibility levels and the costs of learning and operation; these factors determine whether the net benefit of adoption exceeds that of traditional farming, which is precisely the condition in the producers' evolutionary equation. Incentive compatibility theory shapes the design of policy instruments, ensuring that differentiated subsidies to producers and service providers, together with central government rewards, align each party's self interest with the collective objective; in our stability analysis, the conditions for the ideal steady state, namely that local governments find active support worthwhile, service providers profit from high quality services once adoption scales up, and producers benefit from enhanced services and subsidies, are exactly the participation and incentive constraints in formal terms. Stakeholder theory justifies the tripartite asymmetric structure, as each actor holds distinct resources and objectives: local governments balance social benefits against fiscal costs, service providers pursue returns on investment, and producers weigh income gains against production risks; their heterogeneous payoff functions are formalized in the payoff matrix, and their strategic interactions are modeled through replicator dynamics. Together, these three theories address technological attractiveness, institutional incentives, and power asymmetry, forming a coherent causal chain that drives the system toward the collaborative equilibrium.

### 2.2 Problem description

As digital technology and food security become increasingly intertwined, local governments, digital agriculture service providers, and grain producers constitute a closely integrated, mutually reinforcing decision-making system. Local governments bear the political responsibility of implementing the national food security strategy, while also undertaking the developmental task of driving the digital transformation of agriculture and enhancing the competitiveness of regional agriculture (18). Their key decision concerns the level of support to be provided for the development of digital agriculture: namely, whether to adopt a “strong support” strategy that involves active investment and targeted subsidies, or a “weak support” strategy constrained by fiscal pressures and implementation costs. As market entities, digital agriculture service providers must make upfront investments in areas such as technological R&D, equipment deployment, and operation and maintenance services. The crux of their decision-making lies in whether to provide “strong services” (high-quality, high-investment, wide-coverage technical services) or “weak services” (basic, low-cost services with limited coverage) (19). Faced with the emergence of digital technology, grain producers must choose between “adopting digital technology” and “maintaining traditional farming practices.” Their decision hinges on a comprehensive assessment of the technology's potential benefits, learning costs, the availability of supporting services, and policy subsidies (20). These three strategies are mutually influential. Specifically, the level of support provided by local authorities affects service providers' market expectations and investment willingness; the quality of services offered by service providers shapes grain producers' adoption decisions and the likelihood of successful implementation; and grain producers' adoption behavior, in turn, influences the performance evaluation of local authorities and the revenue streams of service providers. This multi-agent, multi-factor, dynamically evolving game process constitutes the core subject of the modeling and analysis in this paper.

### 2.3 Basic assumptions and parameter descriptions

#### 2.3.1 Assumption 1: key stakeholders

The model involves three key stakeholders: local governments, digital agriculture service providers, and grain producers. All three parties are boundedly rational decision-makers. Their strategic choices are not immediately optimal but are gradually adjusted through observation, learning, and trial and error during the dynamic game, ultimately converging on an evolutionarily stable strategy. Decision-making for all parties involves balancing multiple objectives. Specifically, local governments must reconcile their political responsibility for food security with fiscal sustainability; service providers must balance initial investment costs against long-term market returns; and grain producers must weigh the expected income gains from adopting new technologies against the associated risks and costs.

#### 2.3.2 Assumption 2: strategy space

Drawing on the work of Higgins and Bryant (21), Croitoru (24) and others, the strategy space for grain producers is {adopt digital technology, traditional farming}, where they choose digital technology adoption with probability *x* and traditional farming with probability 1−*x*. The adoption of digital technologies refers to the use of such technologies as smart irrigation, precision fertilization, drone-based crop protection, and data monitoring in grain producers' agricultural operations. Traditional farming refers to maintaining existing production methods with little or no use of digital technology. The strategic space for digital agriculture service providers is {strong service, weak service}, where they choose strong service with probability *y* and weak service with probability 1−*y*. “Strong service” refers to high-investment, high-quality, wide-ranging, and responsive digital technology services that entail substantial research and development as well as operation and maintenance inputs. “Weak service” refers to basic, low-cost digital technology services with limited functionality. The local government's strategic options are {strong support, weak support}, with a probability of *z* for strong support and 1−*z* for weak support. “Strong support” refers to local governments actively investing in digital agriculture infrastructure, establishing dedicated subsidy funds, and organizing technical training and extension programs. “Weak support” refers to situations where, due to financial constraints or a lack of prioritization, only basic or token support is provided.

#### 2.3.3 Assumption 3: digital empowerment and the probability of success

Agricultural production is subject to uncertainties, including natural disasters, pests and diseases, and operational management. Consequently, there is a risk of crop failure regardless of whether digital technologies are adopted. Let β_1_(0 <β_1_ <1) denote the probability of success when grain producers adopt digital technologies, and let β_2_(0 <β_2_ <1) denote the probability of success with traditional farming methods. Given the advantages of digital technology in precision management, intelligent decision-making, and risk early warning—which can effectively enhance control over production processes and resilience to risks—it is assumed that β_1_>β_2_ (22). Furthermore, let *d* denote the empowerment coefficient of digital technology on food production (*d* > 1). The larger the value of *d*, the more significant the impact of digital technology on yield and efficiency. Together with the probability of success, it determines the expected output level after the adoption of digital technology (23). Furthermore, let α_1_ and α_2_ denote the accessibility coefficients for grain producers to obtain digital technology resources under strong and weak service conditions, respectively, where 0 <α_1_ <α_2_ <1. A smaller coefficient indicates greater technological accessibility and lower actual costs incurred.

#### 2.3.4 Assumption 4: cost parameters

When local governments opt for strong support, they incur costs *C*_*p*_(*C*_*p*_ > 0), including expenditures on infrastructure development, subsidies, staffing, and organizational costs. The cost of choosing “weak support” is normalized to zero. The cost for a digital agriculture service provider to offer a strong service is *C*_*s*_, while the cost for offering a weak service is *C*_*w*_, with *C*_*s*_ > *C*_*w*_ > 0. The production cost for grain producers adopting digital technology is *C*_*d*_ (which includes basic production costs as well as additional costs associated with learning the technology and using the equipment), while the production cost for traditional farming is *C*_*t*_. Typically, *C*_*d*_ > *C*_*t*_ > 0, reflecting the higher initial investment threshold associated with technology adoption.

#### 2.3.5 Assumption 5: revenue and subsidy parameters

When grain producers adopt digital technology and implement it successfully, their base sales revenue is *R*_*b*_, and their actual revenue following digital transformation is *d*×*R*_*b*_. The base revenue for digital agriculture service providers offering strong service is *R*_*s*_, while that for those offering weak service is *R*_*w*_, with *R*_*s*_ > *R*_*w*_ > 0. When grain producers adopt digital technologies and achieve success, service providers gain additional benefits—such as brand premiums and data value-added services. These additional benefits are denoted as Δ*R*_*s*_ under strong service provision and as Δ*R*_*w*_ under weak service provision, with Δ*R*_*s*_ > Δ*R*_*w*_ > 0. When local governments provide strong support, they offer substantial subsidies *S*_*sa*_ to grain producers who adopt digital technologies and modest subsidies *S*_*wa*_ to those who continue with traditional farming methods. They also provide substantial subsidies *S*_*ss*_ to strong service providers and modest subsidies *S*_*sw*_ to weak service providers. When local government support is weak, a standard subsidy of *S*_*wa*_ and *S*_*sw*_ is provided. The comprehensive social benefits derived by local governments from food security are as follows: high benefits *W*_*h*_ are achieved when digital technologies are successfully adopted, and basic benefits *W*_*l*_ are achieved when traditional farming methods are successful, with *W*_*h*_ > *W*_*l*_ > 0. The central government awards an incentive *A* to local governments based on their performance in promoting digital agriculture and ensuring food security. This incentive is triggered when local governments provide strong support and grain producers successfully adopt digital technologies, provided that the condition *A*>*S*_*sa*_−*S*_*wa*_ is met.

### 2.4 Construction of the payment matrix

Based on the above assumptions, a three-party game payoff matrix is constructed. The three entries in each cell correspond, from top to bottom, to the payoffs for grain producers, digital agriculture service providers, and local governments, respectively.

Using the payoff matrix in Table 1, let *E*_*x*_ denote the expected return for a grain producer who chooses to adopt digital technology, and let *E*_1−*x*_ denote the expected return for choosing traditional farming. Let the replicator dynamics equation be denoted by *F*(*x*). The expected returns under different strategy combinations and the resulting replicator dynamics equation for grain producers are presented in Equation 1:

**Table 1:** Payoff matrix for the three-player game.

Portfolio of strategies	Digital service provider strong service (*y*)		Digital service provider weak service (1 – *y*)	
	Strong support from local government (*z*)	Weak support from local government (1 – *z*)	Strong support from local government (*z*)	Weak support from local government (1 – *z*)
Adoption of digital technology (*x*)	β_1_*dR*_*b*_ – α_1_*C*_*d*_ + *S*_*sa*_	β_1_*dR*_*b*_ – α_1_*C*_*d*_ + *S*_*wa*_	β_1_*dR*_*b*_ – α_2_*C*_*d*_ + *S*_*sa*_	β_1_*dR*_*b*_ – α_2_*C*_*d*_ + *S*_*wa*_
	*R*_*s*_ + β_1_Δ*R*_*s*_ – *C*_*s*_ + *S*_*ss*_	*R*_*s*_ + β_1_Δ*R*_*s*_ – *C*_*s*_ + *S*_*sw*_	*R*_*w*_ + β_1_Δ*R*_*w*_ – *C*_*w*_ + *S*_*sw*_	*R*_*w*_ + β_1_Δ*R*_*w*_ – *C*_*w*_ + *S*_*sw*_
	β_1_*W*_*h*_ + *A* – *C*_*p*_ – *S*_*sa*_ – *S*_*ss*_	β_1_*W*_*h*_ – *S*_*wa*_ – *S*_*sw*_	β_1_*W*_*h*_ + *A* – *C*_*p*_ – *S*_*sa*_ – *S*_*sw*_	β_1_*W*_*h*_ – *S*_*wa*_ – *S*_*sw*_
Using traditional technology (1 – *x*)	β_2_*R*_*b*_ – *C*_*t*_ + *S*_*wa*_	β_2_*R*_*b*_ – *C*_*t*_ + *S*_*wa*_	β_2_*R*_*b*_ – *C*_*t*_ + *S*_*wa*_	β_2_*R*_*b*_ – *C*_*t*_ + *S*_*wa*_
	*R*_*s*_ – *C*_*s*_ + *S*_*ss*_	*R*_*s*_ – *C*_*s*_ + *S*_*sw*_	*R*_*w*_ – *C*_*w*_ + *S*_*sw*_	*R*_*w*_ – *C*_*w*_ + *S*_*sw*_
	β_2_*W*_*l*_ – *C*_*p*_ – *S*_*wa*_ – *S*_*ss*_	β_2_*W*_*l*_ – *S*_*wa*_ – *S*_*sw*_	β_2_*W*_*l*_ – *C*_*p*_ – *S*_*wa*_ – *S*_*sw*_	β_2_*W*_*l*_ – *S*_*wa*_ – *S*_*sw*_

Expected payoff for a grain producer when adopting digital technology:


$$\begin{array}{l} E_{x}=yz(\beta_{1}dR_{b}-\alpha_{1}Cd+S_{sa})+y(1-z)(\beta_{1}dR_{b}-\alpha_{1}C_{d}+S_{wa})\\ \;+(1-y)z(\beta_{1}dR_{b}-\alpha_{2}Cd+S_{sa})\\ \;+\text{(}1-y\text{)(1}-z)(\beta_{1}dR_{b}-\alpha_{2}Cd+S_{wa}\text{)} \end{array}$$

which simplifies to:


$$\begin{array}{l} E_{x}=\beta_{1}dR_{b}-y\alpha_{1}C_{d}-\left(1-y\right)\alpha_{2}C_{d}+zS_{sa}+\left(1-z\right)S_{wa} \end{array}$$

Expected payoff for grain producers when using traditional farming:


$$\begin{array}{l} {\;E}_{1-x}=yz\left(\beta_{2}R_{b}-C_{t}+S_{wa}\right)+y\left(1-z\right)\left(\beta_{2}R_{b}-C_{t}+S_{wa}\right)\\ +\left(1-y\right)z\left(\beta_{2}R_{b}-C_{t}+S_{wa}\right)+\left(1-y\right)\left(1-z\right)\left(\beta_{2}R_{b}-C_{t}+S_{wa}\right) \end{array}$$

Simplified to:


$$\begin{array}{l} E_{1-x}=\beta_{2}R_{b}-C_{t}+S_{wa} \end{array}$$

The replicator dynamics equation for grain producers is thus:


$$\begin{array}{l} \;F(x)=\frac{dx}{dt}=x(1-x)[E_{x}-\bar{E}]=x(1-x)(E_{x}-E_{1-x})\\ =x(1-x)[(\beta_{1}d-\beta_{2})R_{b}-(\alpha_{2}C_{d}-C_{t})+y(\alpha_{2}-\alpha_{1})C_{d}\\ \;+z(S_{sa}-S_{wa})] \end{array}$$ (1)

Similarly, the replicator dynamics equation for the service provider, denoted as *F*(*y*) , is derived as Equation 2.

Expected payoff for a service provider choosing strong service:


$$\begin{array}{l} E_{y}=xz\left(R_{s}+\beta_{1}\Delta R_{s}-C_{s}+S_{ss}\right)+x\left(1-z\right)\left(R_{s}+\beta_{1}\Delta R_{s}-C_{s}+S_{sw}\right)\\ \;+\left(1-x\right)z\left(R_{s}-C_{s}+S_{ss}\right)+\left(1-x\right)\left(1-z\right)\left(R_{s}-C_{s}+S_{sw}\right) \end{array}$$

Simplified to:


$$\begin{array}{l} E_{y}=R_{s}-C_{s}+x\beta_{1}\Delta R_{s}+zS_{ss}+\left(1-z\right)S_{sw} \end{array}$$

Expected payoff for a service provider choosing weak service:


$$\begin{array}{l} {\;E}_{1-y}=xz\left(R_{w}+\beta_{1}\Delta R_{w}-C_{w}+S_{sw}\right)\\ \;+x\left(1-z\right)\left(R_{w}+\beta_{1}\Delta R_{w}-C_{w}+S_{sw}\right)\\ +\left(1-x\right)z\left(R_{w}-C_{w}+S_{sw}\right)+\left(1-x\right)\left(1-z\right)\left(R_{w}-C_{w}+S_{sw}\right) \end{array}$$

Simplified to:


$$\begin{array}{l} E_{1-y}=R_{w}+x\beta_{1}\Delta R_{w}-C_{w}+S_{sw} \end{array}$$

The replicator dynamics equation for service providers is therefore:


$$\begin{array}{l} \;F\left(y\right)=\frac{dy}{dt}=y\left(1-y\right)\left[E_{y}-\bar{E}\right]=y\left(1-y\right)\left(E_{y}-E_{1-y}\right)\\ =y\left(1-y\right)\left[\left(R_{s}-R_{w}\right)-\left(C_{s}-C_{w}\right)+x\beta_{1}\left(\Delta R_{s}-\Delta R_{w}\right)+z\left(S_{ss}-S_{sw}\right)\right] \end{array}$$ (2)

Similarly, the replicator dynamics equation for local governments can be derived as Equation 3.

Expected payoff for local governments choosing strong support:


$$\begin{array}{l} {\;E}_{z}=xy\left(\beta_{1}W_{h}+A-C_{p}-S_{sa}-S_{ss}\right)\\ \;+x\left(1-y\right)\left(\beta_{1}W_{h}+A-C_{p}-S_{sa}-S_{sw}\right)\\ \;+\left(1-x\right)y\left(\beta_{2}W_{l}-C_{p}-S_{wa}-S_{ss}\right)\\ +\left(1-x\right)\left(1-y\right)\left(\beta_{2}W_{l}-C_{p}-S_{wa}-S_{sw}\right) \end{array}$$

Simplified to:


$$\begin{array}{l} E_{z}=x\left(\beta_{1}W_{h}+A-C_{p}-S_{sa}\right)+\left(1-x\right)\left(\beta_{2}W_{l}-C_{p}-S_{wa}\right)\\ \;-S_{sw}-y\left(S_{ss}-S_{sw}\right) \end{array}$$

Expected payoff for local governments choosing weak support:


$$\begin{array}{l} {\;E}_{1-z}=xy\left(\beta_{1}W_{h}-S_{wa}-S_{sw}\right)+x\left(1-y\right)\left(\beta_{1}W_{h}-S_{wa}-S_{sw}\right)\\ +\left(1-x\right)y\left(\beta_{2}W_{l}-S_{wa}-S_{sw}\right)+\left(1-x\right)\left(1-y\right)\left(\beta_{2}W_{l}-S_{wa}-S_{sw}\right) \end{array}$$

Simplified to:

*E*_1−*z*_ = *xβ*_1_*W*_*h*_+(1−*x*)β_2_*W*_*l*_−*S*_*wa*_−*S*_*sw*_

The replicator dynamics equation for local governments is thus:


$$\begin{array}{l} F\left(z\right)=\frac{dy}{dt}=z\left(1-z\right)\left[E_{z}-\bar{E}\right]=z\left(1-z\right)\left(E_{z}-E_{1-z}\right)\\ \;=z\left(1-z\right)\left[xA-C_{p}-x\left(S_{sa}-S_{wa}\right)-y\left(S_{ss}-S_{sw}\right)\right] \end{array}$$ (3)

### 2.5 Analysis of gradual stability

When the system's replicator dynamics equations are set to zero, *x*, *y*, and *z* no longer evolve over time. At this point, all participants have adopted their respective evolutionarily stable strategies. According to the stability theory of differential equations, an equilibrium point is considered stable if the system's replicator dynamics equation and its first derivative both satisfy specific conditions (namely, *F*(*x, y, z*) = 0 and *dF*/*dt* <0). This indicates that the system has reached a stable state. Based on this theoretical framework, the strategic stability analysis of the three parties proceeds as follows:

Taking the derivative of the replicator dynamics Equation 1 for grain producers with respect to*x*yields:


$$\begin{array}{l} F^{\prime }(x)=(1-2x)[(\beta_{1}d-\beta_{2})R_{b}-(\alpha_{2}C_{d}-C_{t})+y(\alpha_{2}-\alpha_{1})C_{d}\\ \;+z(S_{sa}-S_{wa})] \end{array}$$ (4)

According to Equation 4, setting *F*(*x*) = 0 yields *x* = 0, *x* = 1, or possible mixed-strategy solutions. To analyze the evolution of grain producers' strategy choices, let:


$$\begin{array}{l} y^{*}=\frac{\left(\beta_{1}d-\beta_{2}\right)R_{b}-\left(\alpha_{2}C_{d}-C_{t}\right)+z\left(S_{sa}-S_{wa}\right)}{\left(a_{1}-a_{2}\right)C_{d}} \end{array}$$

**Figure 1:**
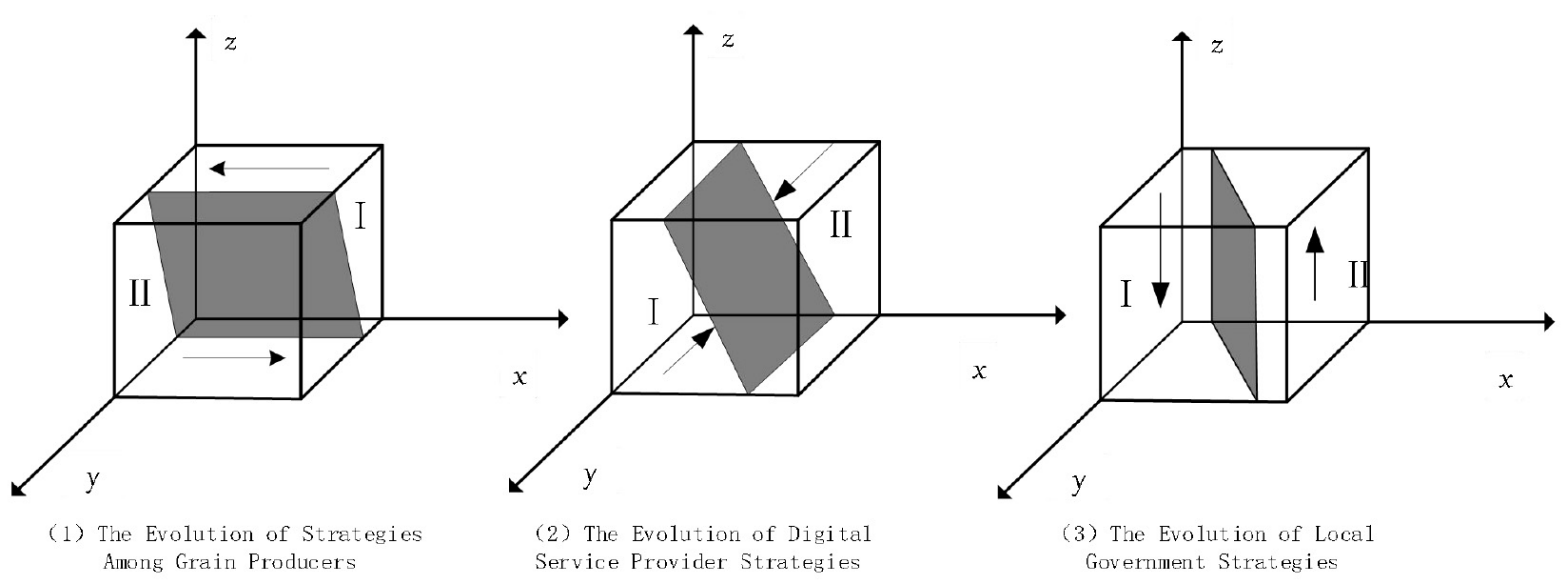
Phases of three-party strategy evolution.

Since α_1_ <α_2_, the denominator is negative. Therefore, the actual meaning of y^*^ must be determined by the sign of the numerator. When *y* = y^*^, *F*(*x*)≡0, and grain producers' strategy choices remain invariant with time, meaning the system can be in a steady state at any *x*. When *y*<y^*^, we have ${F^{\prime }\left(x\right)|}_{x=0}<0$, so *x* = 0 is an evolutionarily stable point, and grain producers tend to choose traditional farming. When *y*>y^*^, ${F^{\prime }\left(x\right)|}_{x=1}<0$, *x* = 1 is an evolutionarily stable point, and grain producers tend to adopt digital technology. As shown in Figure 1(1), the critical plane divides the strategy space into two regions, I and II. Points on the plane remain fixed along the *x*-axis. When grain producers are initially in region I, *x* = 0 is the stable point, and traditional farming is adopted; when in region II, digital technology is adopted.

Similarly, differentiating the service provider's replicator dynamics Equation 2 with respect to *y* yields:


$$\begin{array}{l} F^{\prime }(y)=(1-2y)[(R_{s}-R_{w})-(C_{s}-C_{w})+x\beta_{1}(\Delta R_{s}-\Delta R_{w})\\ \;+z(S_{ss}-S_{sw})] \end{array}$$ (5)

According to Equation 5, Setting the expression in square brackets equal to 0 yields the critical values of *x*:


$$\begin{array}{l} x^{*}=\frac{\left(R_{w}-C_{w}\right)-\left(R_{s}-C_{s}\right)-z\left(S_{ss}-S_{sw}\right)}{\beta_{1}\left(\Delta R_{s}-\Delta R_{w}\right)} \end{array}$$

When *x* = x^*^, *F*(*y*)≡0, and service providers' strategy choices remain invariant with time. When *x*<x^*^, ${F^{\prime }\left(y\right)|}_{y=0}<0$, so *y* = 0 is an evolutionarily stable point, and service providers tend to choose weak service. When *x*>x^*^,${F^{\prime }\left(y\right)|}_{y=1}<0$, so *y* = 1 is an evolutionarily stable point, and they tend to choose strong service. As shown in Figure 1(2), the critical plane *x* = x^*^ divides the strategy space into two regions, I and II. Points on the plane remain stable along the *y*-axis. When service providers are initially in region I, *y* = 0 is the stable point, and weak service is chosen; when in region II, strong service is chosen.

Similarly, differentiating the local government's replicator dynamics Equation 3 with respect to *z* yields:


$$\begin{array}{l} F^{\prime }\left(z\right)=\left(1-2z\right)\left[xA-C_{p}-x\left(S_{sa}-S_{wa}\right)-y\left(S_{ss}-S_{sw}\right)\right] \end{array}$$ (6)

According to Equation 6, setting the expression in square brackets equal to 0 yields:


$$\begin{array}{l} x^{*}=\frac{C_{p}+y\left(S_{ss}-S_{sw}\right)}{A-S_{sa}+S_{wa}} \end{array}$$

When *x* = x^*^, *F*(*z*)≡0, and local governments' strategy choices remain invariant with time. When *x*<x^*^, ${F^{\prime }\left(z\right)|}_{z=0}<0$, so *z* = 0 is an evolutionarily stable point, and local governments tend to adopt weak support. When, ${F^{\prime }\left(z\right)|}_{z=1}<0$, so *z* = 1 is an evolutionarily stable point, and they tend to adopt strong support. As shown in Figure 1(3), the critical plane *x* = x^*^ divides the strategy space into two regions, I and II. Points on the plane remain stable along the *Z*-axis. When a local government is initially in region I, *z* = 0 is the stable point, and weak support is selected; when in region II, strong support is selected.

### 2.6 Analysis of the stability of equilibrium points in a three-party evolutionary game

According to the stability theory of differential equations, when all replicator dynamics equations are zero, the entire dynamic system tends toward a steady state. Solving the system of Equations 1–3 yields eight equilibrium points: *E*_1_(0, 0, 0), *E*_2_(0, 0, 1), *E*_3_(0, 1, 0), *E*_4_(1, 0, 0), *E*_5_(1, 1, 0), *E*_6_(1, 0, 1), *E*_7_(0, 1, 1), and *E*_8_(1, 1, 1). Let *J* denote the Jacobian matrix of the dynamic system comprising grain producers, digital service providers, and local governments. Let λ_*i*_(*i*∈{1, 2, 3}) denote the eigenvalues of *J*. The eigenvalues associated with the eight equilibrium points are shown in Table 2. The equilibrium solutions obtained from the replicator dynamics equations are not necessarily evolutionarily stable. Only when they constitute pure-strategy Nash equilibria and satisfy local stability conditions can they qualify as evolutionarily stable strategies (ESSs). Therefore, eigenvalue analysis of the system's Jacobian matrix is used to determine the stability of each equilibrium point. If all eigenvalues have negative real parts, the equilibrium point is asymptotically stable and represents an ESS. If at least one eigenvalue has a positive real part, the point is unstable. If the eigenvalues have mixed signs (some positive, some negative), the point is a saddle point. Consequently, the Jacobian matrix constructed in this way can be expressed as:


$$J=\{\begin{array}{ccc} \frac{\partial F(x)}{\partial x}\; & \frac{\partial F(x)}{\partial y}\; & \frac{\partial F(x)}{\partial z}\\ \frac{\partial F(y)}{\partial x}\; & \frac{\partial F(y)}{\partial y}\; & \frac{\partial F(y)}{\partial z}\\ \frac{\partial F(z)}{\partial x}\; & \frac{\partial F(z)}{\partial y} & \frac{\partial F(z)}{\partial z} \end{array}=[\begin{array}{ccc} J_{11}\; & J_{12} & J_{13}\\ J_{21} & J_{22} & J_{23}\\ J_{31} & J_{32} & J_{33} \end{array}]$$

**Table 2:** Eigenvalues corresponding to the equilibrium points.

Equilibrium point	λ_1_	λ_2_	λ_3_
*E*_1_	(β_1_*d*−−β_2_)*R*_*b*_ – (α_2_*C*_*d*_ −−*C*_*t*_)	(*R*_*s*_ – *C*_*s*_) – (*R*_*w*_ – *C*_*w*_)	–*C*_*p*_
*E*_2_	(β_1_d – β_2_)*R*_*b*_ – (α_2_*C*_*d*_ – *C*_*t*_) + (*S*_*sa*_ – *S*_*wa*_)	(*R*_*s*_ – *C*_*s*_) – (*R*_*w*_ – *C*_*w*_) + (*S*_*ss*_ – *S*_*sw*_)	*C*_*p*_
*E*_3_	(β_1_d – β_2_)*R*_*b*_ – (α_1_*C*_*d*_ – *C*_*t*_)	–[(*R*_*s*_ – *C*_*s*_) – (*R*_*w*_ – *C*_*w*_)]	–*C*_*p*_ – (*S*_*ss*_ – *S*_*sw*_)
*E*_4_	–[(β_1_d – β_2_)*R*_*b*_ – (α_2_*C*_*d*_ – *C*_*t*_)]	(*R*_*s*_ – *C*_*s*_) – (*R*_*w*_ – *C*_*w*_) + β_1_(Δ*R*_*s*_ – Δ*R*_*w*_)	*A* – *S*_*sa*_ + *S*_*wa*_ – *C*_*p*_
*E*_5_	–[(β_1_d – β_2_)*R*_*b*_ – (α_1_*C*_*d*_ – *C*_*t*_)]	–[(*R*_*s*_ – *C*_*s*_) – (*R*_*w*_ – *C*_*w*_)] – β_1_(Δ*R*_*s*_ – Δ*R*_*w*_)	*A* – S_*sa*_ + S_*wa*_ – *C*_*p*_ – (*S*_*ss*_ – *S*_*sw*_)
*E*_6_	–[(β_1_d – β_2_)*R*_*b*_ – (α_2_*C*_*d*_ – *C*_*t*_)] – (*S*_*sa*_ – *S*_*wa*_)	(*R*_*s*_ – *C*_*s*_) – (*R*_*w*_ – *C*_*w*_) + β_1_(Δ*R*_*s*_ – Δ*R*_*w*_) + (*S*_*ss*_ – *S*_*sw*_)	*C*_*p*_ – (*A* – S_*sa*_ + *S*_*wa*_)
*E*_7_	(β_1_d – β_2_)*R*_*b*_ – (α_1_*C*_*d*_ – *C*_*t*_) + (*S*_*sa*_ – *S*_*wa*_)	–[(*R*_*s*_ – *C*_*s*_) – (*R*_*w*_ – *C*_*w*_)] – (*S*_*ss*_ – *S*_*sw*_)	*C*_*p*_ + (*S*_*ss*_ – *S*_*sw*_)
*E*_8_	–[(β_1_d – β_2_)*R*_*b*_ – (α_1_*C*_*d*_ – *C*_*t*_)] – (*S*_*sa*_ – *S*_*wa*_)	–[(*R*_*s*_ – *C*_*s*_) – (*R*_*w*_ – *C*_*w*_)] – (*S*_*ss*_ – *S*_*sw*_) – β_1_(Δ*R*_*s*_ – Δ*R*_*w*_)	*C*_*p*_ + (*S*_*ss*_ – *S*_*sw*_) – (A – *S*_*sa*_ + *S*_*wa*_)

Specifically:


$$\begin{array}{l} J_{11}=(1-2x)[(\beta_{1}d-\beta_{2})R_{b}-(\alpha_{2}C_{d}-C_{t})+y(\alpha_{2}-\alpha_{1})C_{d}\\ \;+z(S_{sa}-S_{wa})]\\ \;J_{12}=-x(1-x)(\alpha_{2}-\alpha_{1})C_{d} \end{array}$$

$$\begin{array}{l} J_{13}=x(1-x)(S_{sa}-S_{wa})\\ J_{21}=y(1-y)\beta_{1}(\Delta R_{s}-\Delta R_{w})\\ J_{22}=(1-2y)[(R_{s}-C_{s})-(R_{w}-C_{w})+x\beta_{1}(\Delta R_{s}-\Delta R_{w})\\ \;+z(S_{ss}-S_{sw})]\\ J_{23}=y(1-y)(S_{ss}-S_{sw})\\ J_{31}=z(1-z)(A-S_{sa}+S_{wa})\\ J_{32}=-z(1-z)(S_{ss}-S_{sw})\\ J_{33}=z(1-2z)[x(A-S_{sa}+S_{wa})-C_{p}-y(S_{ss}-S_{sw})] \end{array}$$

Let *E* = (β_1_*d*−β_2_)*R*_*b*_−(α_2_*C*_*d*_−*C*_*t*_) and *F* = (*R*_*s*_−*C*_*s*_)−(*R*_*w*_−*C*_*w*_). Given the numerous factors influencing the system's evolutionary stability and the rather complex mathematical relationships among the parameters, this paper examines the stability of the system's evolution under the following four scenarios, as summarized in Table 3.

**Table 3:** Eigenvalue sign patterns of the Jacobian matrix at each equilibrium point.

Balance	*E* > 0, *F* > 0		*E* > 0, *F*<0		*E*<0, *F* > 0		*E*<0, *F*<0	
	λ	Stability	λ	Stability	λ	Stability	λ	Stability
*E*_1_	(+, +, –)	Unstable	(+, –, –)	Unstable	(–, +, –)	Unstable	(–, –, –)	ESS
*E*_2_	(+, +, +)	Unstable	(+, ^*^, +)	Unstable	(^*^, +, +)	Unstable	(^*^, ^*^, +)	Unstable
*E*_3_	(+, –, –)	Unstable	(+, +, –)	Unstable	(^*^, –, –)	To be discussed	(^*^, +, –)	Unstable
*E*_4_	(–, +, ^*^)	Unstable	(–, ^*^, ^*^)	To be discussed	(+, +, ^*^)	Unstable	(+, ^*^, ^*^)	Unstable
*E*_5_	(–, –, ^*^)	To be discussed	(–, ^*^, ^*^)	To be discussed	(^*^, –, ^*^)	To be discussed	(^*^, ^*^, ^*^)	To be discussed
	(–, +, ^*^)	Unstable	(–, ^*^, ^*^)	To be discussed	(^*^, +, ^*^)	Unstable	(^*^, ^*^, ^*^)	To be discussed
*E*_7_	(+,–,+)	Unstable	(+,^*^, +)	Unstable	(^*^,–, +)	Unstable	(–, ^*^, +)	Unstable
*E*_8_	(–, –, ^*^)	To be discussed	(–, ^*^, ^*^)	To be discussed	(^*^, –, ^*^)	To be discussed	(^*^, ^*^, ^*^)	To be discussed

Since α_1_ <α_2_, if *E* > 0, then (β_1_*d* –*t*β_2_)R_*b*_ – (α_1_C_*d*_ – C_*t*_) is necessarily “+.” If *E* <0, the sign of this expression is undetermined and is denoted by “^*^.”

*Scenario 1*: When *E*>0 and *F*>0, define *G* = *A*−*C*_*p*_−(*S*_*ss*_−*S*_*sw*_)−(*S*_*sa*_−*S*_*wa*_). If *G*>0 the equilibrium point E8(1, 1, 1) is the ESS of the system; if *G* <0, E5(1, 1, 0) is the ESS. According to the eigenvalue analysis, *E*>0 means that, even with weak service provision and no additional subsidies, grain producers obtain a higher payoff from adopting digital technology than from traditional farming. *F*>0 means that, absent technology adoption by grain producers and without additional subsidies, strong service yields a higher base payoff for service providers than weak service. *G*>0 means that, when local governments choose strong support, the central government incentive is sufficient to cover the total additional expenditures—namely, the increased subsidy outlays and the extra administrative costs.

*Scenario 2*: When *E*>0 and *F* <0, define *H* = *F*+β_1_(Δ*R*_*s*_−Δ*R*_*w*_) = (*S*_*ss*_−*S*_*sw*_). If *H*>0 the equilibrium points E5(1, 1, 0) and E8(1, 1, 1) are candidate ESSs. In this sub-case, if *G*>0, E8(1, 1, 1) is the ESS; if *G* <0, E5(1, 1, 0) is the ESS. If instead *H* <0, the equilibrium points E4(1, 0, 0) and E6(1, 0, 1) become candidate ESSs. Under this condition, E6 (1, 0, 1) is the ESS, when *G*>0; Otherwise, E4(1, 0, 0) is the ESS. As the eigenvalue analysis in Tables 2, 3 shows, *E*>0 implies that, under weak service provision and without additional subsidies, grain producers obtain a higher payoff from adopting digital technology than from traditional farming. Consequently, grain producers have an endogenous incentive to adopt the technology. *F* <0 indicates that, absent technology adoption and without additional subsidies, the base payoff for strong service is lower than that for weak service. In other words, strong service does not offer a profit advantage at the basic business-model level, and service providers will tend to choose weak service unless additional incentives are present. If, the total payoff for strong service exceeds that for weak service when grain producers adopt digital technology and local governments provide strong support. In this scenario, driven by the dual incentives of producer adoption and government subsidies, service providers will switch to providing strong service.

*Scenario 3*: When *E* <0 and *F*>0, define *I* = (β_1_*d*−β_2_)*R*_*b*_−(α_1_*C*_*d*_−*C*_*t*_). If *I*>0 the net benefit to grain producers under strong service and strong government support exceeds that of traditional farming, thereby stimulating their willingness to adopt digital technology. In this case, if *G*>0, E8(1, 1, 1) is the ESS; if *G* <0 local governments tend toward weak support, but the system may still converge to E5(1, 1, 0) because grain producers have adopted the technology and service providers continue to offer strong service. If *I* <0, then even with strong service and government subsidies, the net benefit of digital technology adoption for grain producers remains lower than that of traditional farming; they will therefore continue to choose traditional farming. In this case, service providers still offer strong service because *F*>0, if *G* <0, the system converges to E3(0, 1, 0). If *G*>0, although local governments are willing to provide strong support, they cannot realize the expected benefits because grain producers do not adopt the technology. Consequently, the system may struggle to stabilize at a pure-strategy equilibrium, and fluctuations or mixed strategies may arise. The condition *I*>0 captures the net benefit gain for grain producers when two improvements occur simultaneously: service providers raise their service quality (reducing the accessibility coefficient to α_1_), and local governments increase their support (offering an incremental subsidy of *S*_*sa*_−*S*_*wa*_). Together, these two improvements make digital technology adoption more profitable than traditional farming.

*Scenario 4*: When *E* <0 and *F* <0, dual market-driven momentum is absent: digital technology has weak intrinsic appeal, and service providers face poor underlying profitability. In the absence of effective policy intervention, the system naturally converges to the low-level equilibrium E1(0, 0, 0), in which all three parties remain in a suboptimal state. If and *H*>0, the equilibrium points E5(1, 1, 0) and E8(1, 1, 1) are candidate ESSs. Under these conditions, E8(1, 1, 1) is the ESS If *G*>0, whereas E5(1, 1, 0) is the ESS if *G* <0. If instead *H* <0 and *G* <0 (provided that the additional net subsidy *E*<*S*_*sa*_−*S*_*wa*_ is satisfied), the equilibrium point E6(1, 0, 1) becomes the ESS.

Table 4 systematically summarizes, across different combinations of digital technology's endogenous appeal and service providers' fundamental profitability, the critical conditions for the system to converge to the ideal steady state E8(1, 1, 1) along with the corresponding policy priorities. When *E*>0 and *F*>0, the market already possesses the endogenous momentum for spontaneous technology adoption and high-quality service provision. In this case, achieving tripartite coordination requires only the optimization of central incentives and the reduction of local implementation costs to ensure *G*>0. When *E*>0 and *F* <0, the technology is viable, but service providers face profitability challenges. Policy should therefore focus on supporting digital service providers and expanding technology adoption to unlock additional benefits, while maintaining government incentives. Targeted subsidies should be designed to ensure that both *H*>0 and *G*>0 are satisfied. When *E* <0 and *F*>0, the supply of services is sufficient, but the technology itself lacks adequate appeal. Stimulating grain producers' willingness to adopt therefore becomes the central task. It is necessary to make the net benefit of technology adoption under enhanced services exceed that of traditional farming (i.e., *I*>0), by improving service accessibility and increasing targeted subsidies to producers, complemented by government incentives. When *E* <0 and *F* <0, the market faces a dual failure—of both technology and services—and the system risks sliding toward the low-level equilibrium E1(0, 0, 0). Only through systemic intervention—namely, simultaneously enhancing technological empowerment, service profitability, and government incentives—can this situation be reversed, provided that *I*>0, *H*>0, and *G*>0 are all satisfied.

**Table 4:** Conditions for the system to converge to E8(1, 1, 1) under the four scenarios.

Situation	Environment	E8 equilibrium conditions	Policy implications
*E* > 0, *F* > 0	Effective technology, profitable services	*G* > 0	Improve central incentives and reduce local implementation costs.
*E* > 0, *F* <0	The technology is effective, but service providers have weak profitability	*H* > 0, *G* > 0	Support service providers through cost subsidies and expand the adoption scale to unlock additional benefits.
*E* <0, *F* > 0	Strong commitment to service, but lacking in technical capability.	*I* > 0, *G* > 0	Lower adoption barriers through demonstration projects, training, and targeted subsidies.
*E* <0, *F* <0	A failure on both the technical and service fronts	*I* > 0, *H* > 0, *G* > 0	Deploy adapted low-cost digital solutions combined with government procurement and coordinated incentives.

To further bridge the theoretical analysis with practical realities, it is useful to consider how the four evolutionary scenarios may correspond to the heterogeneous conditions across China's major grain-producing regions. For instance, the Northeast Plain, characterized by large-scale contiguous farmland and strong service supply capacity, might be interpreted as a case where service providers have sufficient momentum (*F*>0) while the technology itself may not yet have achieved universal appeal among producers (*E* <0), corresponding roughly to Scenario 3, where the policy priority lies in stimulating producers' willingness to adopt through demonstration projects and targeted subsidies. The Yangtze River Basin, with its medium-sized land holdings, dense service networks, and documented positive effects of digitalization on food security, appears closer to Scenario 2, where the technology is effective (*E*>0) but service providers face profitability challenges (*F* <0), suggesting that support for service providers through cost subsidies and expansion of adoption scale may be particularly relevant. In contrast, the mountainous areas of Southwest China, dominated by fragmented smallholdings and low service provider density, likely reflect Scenario 4, where both technological appeal and service profitability are weak (*E* <0, *F* <0), and systemic intervention involving adapted low-cost digital solutions, government procurement, and coordinated incentives may be necessary to avoid a low-level equilibrium. These examples are not intended as definitive classifications but rather as illustrative mappings that highlight the need for regionally differentiated policy designs, as the same policy instrument may yield quite different outcomes depending on local conditions in land scale, infrastructure, and market development.

## 3 Simulation analysis

### 3.1 Assigning numerical values

This paper adopts a multi-source data fusion approach to parameter calibration, ensuring the scientific validity and reliability of the simulation results. In October 2025, the research team conducted field surveys in selected areas of Chongqing, China. Using stratified sampling, they obtained a representative sample of farming households and collected key indicators, such as production costs and revenues. Simultaneously, government portals and authoritative media outlets were searched to retrieve relevant policy documents, government transparency information, and literature on evolutionary game theory. Building upon the above data, the expert consultation method was further employed. In-depth interviews were conducted with staff members of the Chongqing Municipal Commission of Agriculture and Rural Affairs to obtain their professional judgment on appropriate values for the model parameters. Through multi-dimensional data collection and cross-validation, the final benchmark parameter values were determined, as shown in Table 5. Calculations show that, under this parameter set, *E* = (0.9 × 1.25 − 0.75) × 1200−(0.9 × 1000 − 800) = 400>0 and *F* = (80 − 70)−(40 − 25) = −5 <0. This indicates that the development of digital agriculture in China's major grain-producing regions is currently characterized by “technological effectiveness but weak underlying profitability among service providers.”

**Table 5:** Key parameter settings and their basis.

Parameter symbols	Parameter descriptions	Reference value	Unit	Main basis and explanation
*R*_*b*_	Basic sales revenue from traditional cultivation	1,200	Yuan/mu	Based on the average output value per mu for rice and wheat nationwide in 2025 (approximately 1,100–1,300 yuan per mu)
*d*	Digital Technology Empowerment Coefficient	1.25	–	Based on data from smart farming initiatives, yields have increased by approximately 10% to 30%
β_1_	Probability of successful adoption of digital technology	0.9	–	Digital technology can reduce disaster losses, as demonstrated by actuarial data from agricultural insurance
β_2_	Success rate of traditional cultivation	0.75	–	Crop production is highly susceptible to natural disasters; refer to historical disaster rates
*C*_*d*_	Production costs associated with the adoption of digital technology	1,000	Yuan/mu	Basic cost of approximately 850 yuan + technical usage fee of 150 yuan
*C*_*t*_	Production costs in traditional farming	850	Yuan/mu	Calculated by combining survey data with the costs of materials and services and labor costs as reported in the provincial agricultural product cost and revenue bulletins
α_1_	Service Accessibility Coefficient	0.75	–	The proportion of actual costs borne under enhanced service provision, based on survey data
α_2_	Weak service accessibility coefficient	0.90	–	Accessibility is poor in areas with poor service provision, and the cost burden is higher
*R*_*s*_	Service providers' core service revenue	80	Yuan/mu	Digital agriculture service fees (including equipment hire, data services, etc.)
*R*_*w*_	Service providers' basic revenue from low-value services	40	Yuan/mu	Basic technical service fee; limited scope of services
*C*_*s*_	High service costs	70	Yuan/mu	including equipment maintenance, staff training and data platform operations, etc.
*C*_*w*_	Low service costs	25	Yuan/mu	Set in accordance with the relevant criteria in the local government's assessment and reward scheme for villages.
Δ*R*_*s*_	Enhanced service and additional benefits	30	Yuan/mu	Brand premium, data monetization, customer retention, etc.
Δ*R*_*w*_	Additional revenue from low-performing services	10	Yuan/mu	Additional benefits
*S*_*sa*_	Heavy subsidies for grain farmers	150	Yuan/mu	Subsidy for the Protection of Arable Land Fertility + Special Subsidy for the Application of Digital Technology
*S*_*wa*_	Basic subsidy for grain producers	100	Yuan/mu	Includes only the subsidy for the protection of soil fertility on conventional arable land
*S*_*ss*_	Heavy subsidies from service providers	40	Yuan/mu	Special Subsidy for Smart Agriculture Services
*S*_*sw*_	Basic subsidy for service providers	15	Yuan/mu	Universal agricultural service subsidies
	The government achieves significant social benefits	2,000	Yuan/mu	Contribution to food security + ecological benefits + benefits for rural social stability
*W*_*l*_	Basic social benefits provided by the government	1,200	Yuan/mu	Contribution to basic food security only
*A*	Central Government Award	200	Yuan/mu	Central Government Incentives for Local Achievements in Food Security and Digital Agriculture
*C*_*p*_	Government-mandated administrative costs	80	Yuan/mu	Additional expenditure on regulation, publicity, and coordination

The practical basis for the above parameter ranges can be substantiated from two perspectives. First, the positive impact of digital technology on staple crop yields and farmer income has been widely verified in China. According to data released by China's Ministry of Agriculture and Rural Affairs, the application of digital agricultural management technologies can increase yields in major grain-producing regions by more than 30% relative to the average. In Qingshen County, Sichuan Province, the use of a digital agriculture platform enabled a rice yield of 800 kg per mu, an increase of approximately 20% over traditional farming methods. Moreover, the Report on the Assessment of the Development Level of Agricultural and Rural Informatization in China's Counties indicates that the informatization rate in agricultural production reached 27.6% in 2023, with technologies such as remote sensing monitoring, precision fertilization, and drone-based crop protection rapidly gaining widespread adoption in large-scale production areas. These data demonstrate that the conditions for technological effectiveness (*E*>0) have a solid practical foundation. On the other hand, the core business model of digital agriculture service providers remains relatively fragile in practice. Unlike cash crops, staple grains generate lower value-added per unit area, making it difficult for service providers to cover the full costs of high-quality services through basic service fees alone. Research indicates that the market for digital services in open-field farming is still in its infancy. Service providers generally face a “diseconomies of scale” dilemma, where the small and fragmented nature of farmland makes it difficult to achieve cost efficiency at higher service volumes. Taking drone-based crop protection as an example, the service fee ranges from approximately 10 to 20 yuan per mu. However, once equipment depreciation, battery wear and tear, and pilot training costs are factored in, profits become extremely slim—or even non-existent—under small-scale, fragmented operations. Leading Chinese companies, such as XAG, remained unprofitable in 2023 and turned a profit only in 2024. Meanwhile, the majority of small and medium-sized service providers continue to rely heavily on government procurement and subsidies. This reality confirms the validity of *F* <0 under the baseline parameters: without additional revenue or policy incentives, service providers find it difficult to sustain a high level of service provision.

### 3.2 Baseline scenario simulation and evolution pathways

**Figure 2:**
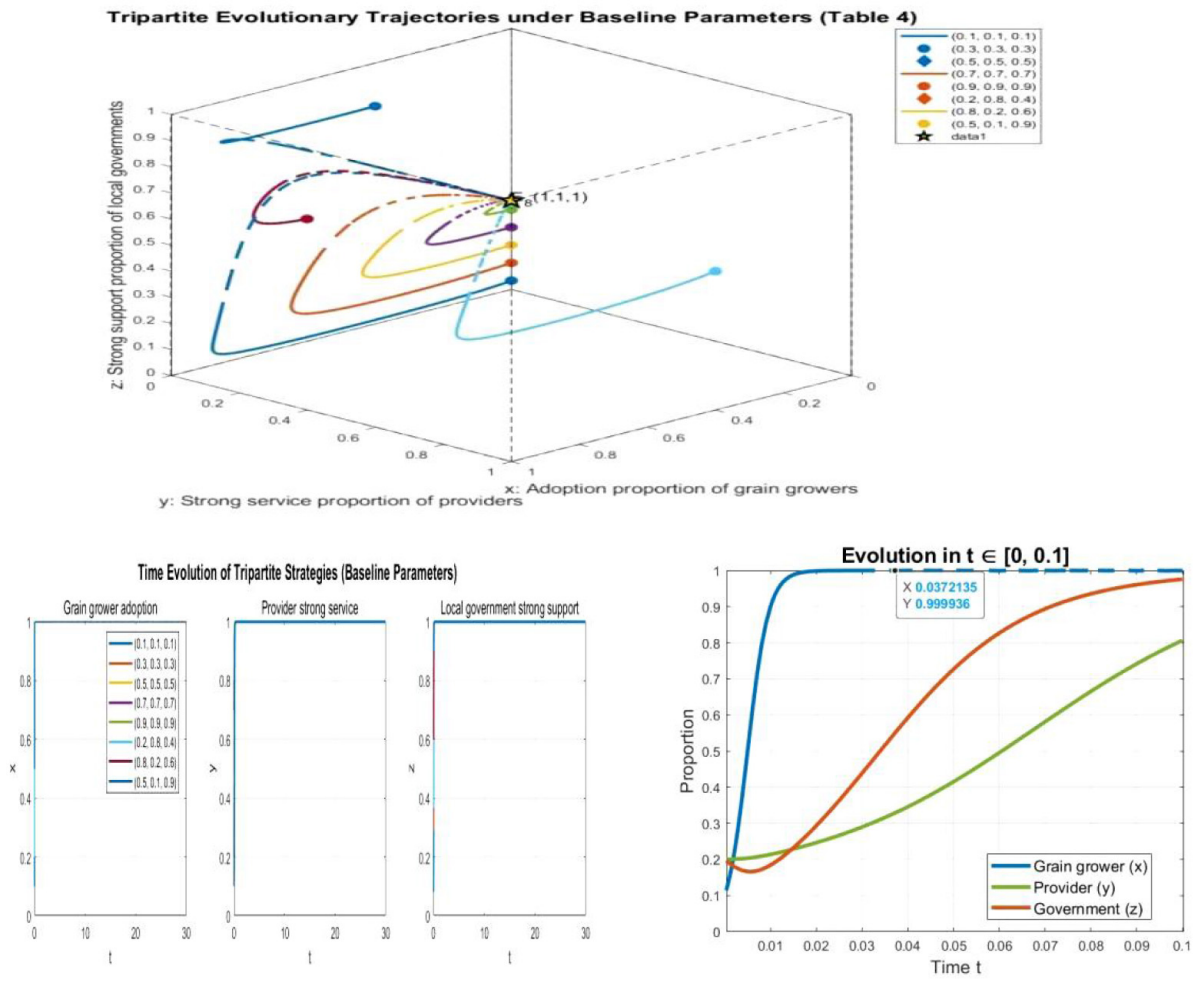
Evolution of the initial paths of the three strategies under the benchmark parameters.

Based on the benchmark parameters given in Table 5, an initial-path analysis of the three-party replicator dynamics equations was performed using MATLAB 2024. Eight representative sets of initial strategy proportions were defined: low (0.1, 0.1, 0.1), medium (0.3, 0.3, 0.3), high-symmetry (0.5, 0.5, 0.5; 0.7, 0.7, 0.7), and very high symmetry (0.9, 0.9, 0.9), together with three asymmetric sets (0.2, 0.8, 0.4), (0.8, 0.2, 0.6), and (0.5, 0.1, 0.9). This design allowed a comprehensive examination of how initial conditions influence the system's evolutionary trajectory. The simulation time was set to *t*∈[0, 30], and the results are shown in Figure 2.

The simulation results show that all eight evolutionary trajectories converge, without exception, to the equilibrium point E8(1, 1, 1). This corresponds to the ideal steady state in which grain producers adopt digital technology, digital agriculture service providers offer strong service, and local governments provide strong support. As shown in the 3D trajectory plot, regardless of the initial strategy proportions within the unit cube, the system exhibits a clear tendency to converge toward E8, and the final positions are highly concentrated at that ideal steady state. This indicates that, under the baseline parameter configuration, the system exhibits global convergence: the ideal steady state exerts universal attraction on all initial states, and there are no multiple equilibria or path bifurcations.

It can be clearly observed from the 2D time-series plot that the three strategy proportions converge at markedly different rates. The adoption rate of grain producers (*x*) converges to 1 first, followed by strong support from local governments (*z*), while the proportion of strong service provision by service providers (*y*) converges the most slowly. This temporal pattern reveals a potential pathway for digital technology to enhance food security. When the economic benefits of the technology are sufficiently significant (*E*>0) and grain producers are responsive to income increases, market demand can surge ahead of policy and supply, giving rise to a “producer-driven” evolutionary pattern. In practice, this corresponds to the rapid uptake of smartphones in agriculture, where grain producers spontaneously purchase equipment such as drones and smart sensors. Their demand then compels local governments to introduce supporting subsidy programs and attracts service providers to accelerate market entry. In the early stages, local governments often lack strong incentives to offer support because of low adoption rates and high costs; only after adoption reaches a critical mass do they become proactive.

### 3.3 Sensitivity analysis of key parameters

#### 3.3.1 Impact of the Digital Empowerment Coefficient (*d*)

**Figure 3:**
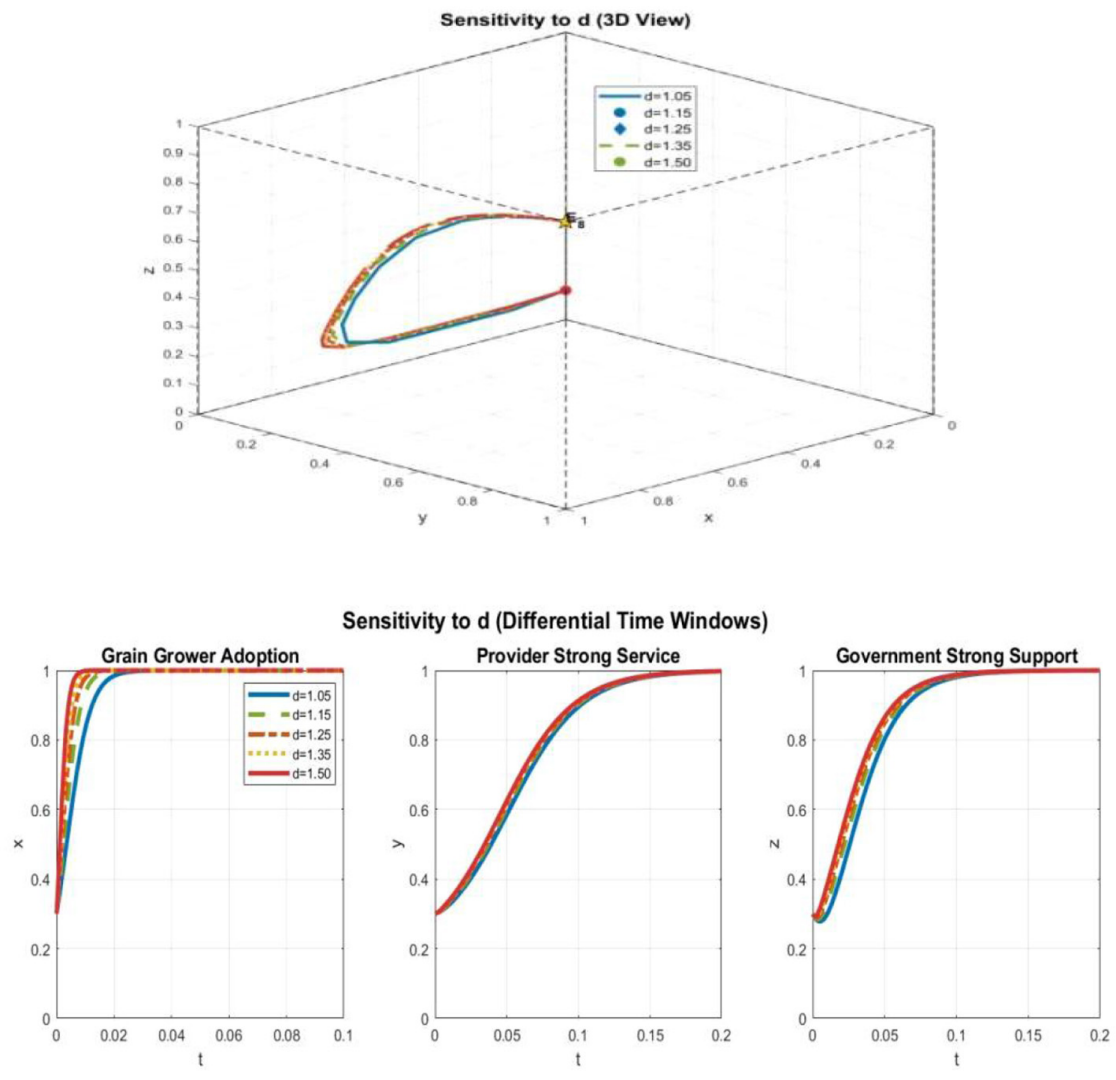
Impact of changes in *d* on the strategies of grain producers, digital service providers, and local governments.

The digital empowerment coefficient *d* captures the extent to which digital technologies increase grain production and improve efficiency; it is the key variable determining grain producers' incentives to adopt these technologies. Taking the baseline value *d*= 1.25 as a reference, simulations were conducted with*d*set to 1.05, 1.15, 1.35, and 1.50. The resulting 3D evolution trajectories and 2D time series are shown in Figure 3.

As shown in the 3D trajectory plot, the system converges to the ideal steady state E8(1, 1, 1) for all tested values of *d*. This indicates that, provided the baseline policy parameters (such as subsidies and central government incentives) remain unchanged, the enabling effect of digital technology alone is sufficient to drive the system toward tripartite coordination. Judging from the 2D evolution plots, the convergence speed of *x*, *y*, and *z* is markedly faster under *d* = 1.5 than under *d* = 1.05. This confirms that the digital empowerment coefficient is the fundamental driving force behind the system's evolution toward the ideal steady state. Its value not only determines the stability of the steady state but also profoundly influences the speed of adoption during the initial implementation stage. At the policy level, investment in the research and development of core digital agriculture technologies should be continuously increased. Measures such as integrating superior crop varieties with best agronomic practices and localizing smart equipment can effectively enhance the enabling effects of these technologies in field production.

#### 3.3.2 Impact of strong subsidies for grain producers (*S*_*sa*_)

**Figure 4:**
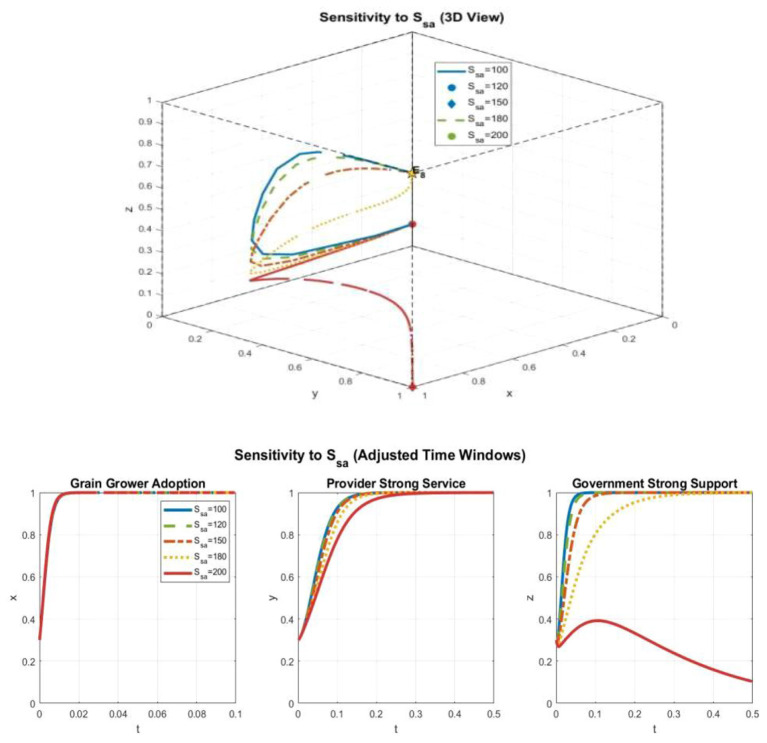
Impact of changes in *S*_*sa*_ on the strategies of grain producers, digital service providers, and local governments.

The strong subsidy *S*_*sa*_ for grain producers is a direct fiscal instrument by which local governments incentivize the adoption of digital technologies. Taking the benchmark value *S*_*sa*_ = 150, simulations were conducted with *S*_*sa*_ set to 100, 120, 180, and 200. The resulting 3D evolution trajectories and 2D early-stage time series are shown in Figure 4.

As can be seen from the 3D trajectory plot, when *S*_*sa*_ ≤ 180, the system ultimately converges to the ideal steady state E8(1, 1, 1), whereas when = 200, it converges to E5(1, 1, 0). This implies that, although strong subsidies for grain producers (*S*_*sa*_) are highly effective in encouraging technology adoption, a higher subsidy is not necessarily better. An excessively high subsidy may increase the government's fiscal burden, leading it to re-evaluate its support strategy. From the 2D dynamics, significantly accelerates both grain producers' adoption of digital technology and service providers' provision of strong service, with grain producers converging at a visibly faster rate. In the 2D plot for local governments, when *S*_*sa*_ = 200, local governments tend to converge toward weak support. This occurs because, in the replicator dynamics equation *F*(*z*) governing local governments, a larger *S*_*sa*_ reduces the term *x*[*A*−(*S*_*sa*_−*S*_*wa*_)], thereby lowering the government's net revenue. This implies that, in the early stages when the adoption rate *x* has not yet risen sufficiently, an excessively high subsidy commitment increases the anticipated fiscal burden and weakens the government's immediate incentive to provide strong support. The simulations suggest that, under the baseline parameters, maintaining *S*_*sa*_ within the range of 150–180 yuan per mu can effectively accelerate technology adoption by grain producers without unduly undermining government incentives. In practice, subsidies could be linked to performance indicators such as actual yield increases and the acreage on which the technology is applied. This would mark a shift from a “universal” approach to a “targeted, performance-based” one, helping to ensure incentive effectiveness while keeping fiscal costs under control.

#### 3.3.3 Impact of service providers' operating costs (*C*_*s*_)

**Figure 5:**
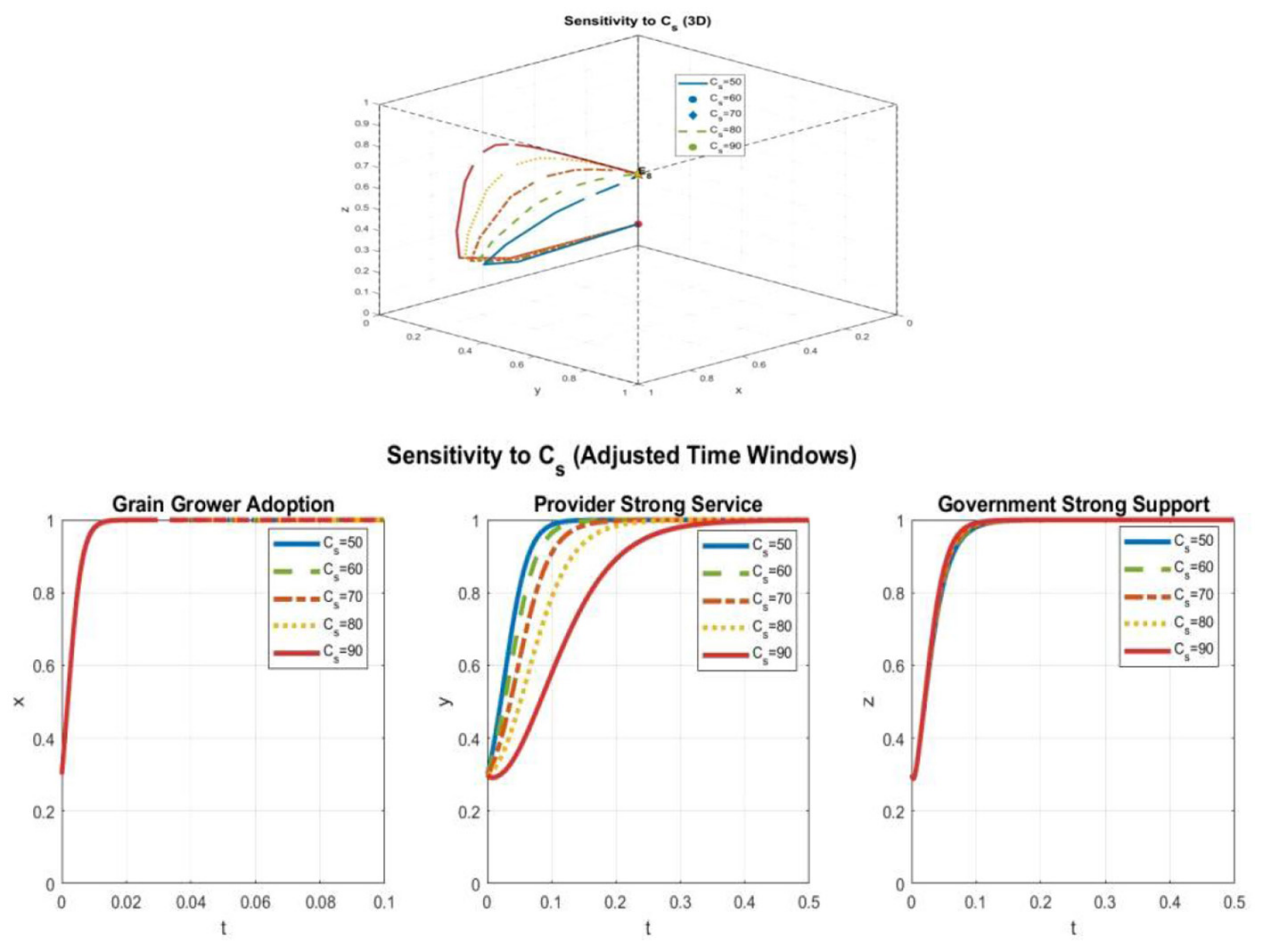
Impact of changes in *C*_*s*_ on the strategic choices of the three parties.

The service provider's cost of providing strong service (*C*_*s*_) is the key variable determining the profitability and service quality on the digital agriculture supply side. Taking *C*_*s*_ = 70 as the benchmark, simulations were conducted with set *C*_*s*_ to 50, 60, 80, and 90 yuan per mu. The resulting 3D evolution trajectories and 2D early-stage time series are shown in Figure 5.

As can be seen from the 3D trajectory plot, the system converges to the ideal steady state E8(1, 1, 1) for all tested values of *C*_*s*_, indicating that—at unchanged baseline policy parameters—variations in service costs do not alter the system's global stability. However, the curvature and speed of the convergence path vary significantly as *C*_*s*_ increases. When *C*_*s*_ = 50, the trajectory extends almost straight toward E8, whereas when *C*_*s*_ = 90, the trajectory veers markedly toward the low-*y* region in the early stages of evolution. The latter exhibits a pattern of first meandering then converging, requiring a longer adjustment period to approach a steady state. This suggests that, although the attraction of the ideal steady state remains, high service costs significantly prolong the system's transition process and increase coordination costs during the initial rollout phase. Judging from the 2D plots, variations in *C*_*s*_ have a pronounced impact on the strategic choices of digital service providers (*y*). When *C*_*s*_ = 50 *y* converges markedly faster than when *C*_*s*_ = 90. This occurs because a larger *C*_*s*_ reduces the profit margin *R*_*s*_−*C*_*s*_ in the service provider's replicator dynamics equation *F*(*y*), thereby lowering their payoff. Further analysis reveals that when *C*_*s*_ drops below 65 yuan per mu, *F* = (*R*_*s*_−*C*_*s*_)−(*R*_*w*_−*C*_*w*_) turns positive, and the system transitions from the “technologically effective but weak provider profitability” regime (*E*>0, *F* <0) to the ideal “technologically effective and service-profitable” regime (*E*>0, *F*>0). At this stage, service providers can offer strong service on their own, without the need for additional government subsidies, and the system's convergence rate improves across the board. Consequently, during the early stages of market expansion, when *x* and *z* have not yet grown sufficiently, service providers' strategic choices depend heavily on their underlying profitability. High costs directly suppress their willingness to expand service provision, resulting in a significant convergence lag.

#### 3.3.4 Impact of central government incentives (*A*)

**Figure 6:**
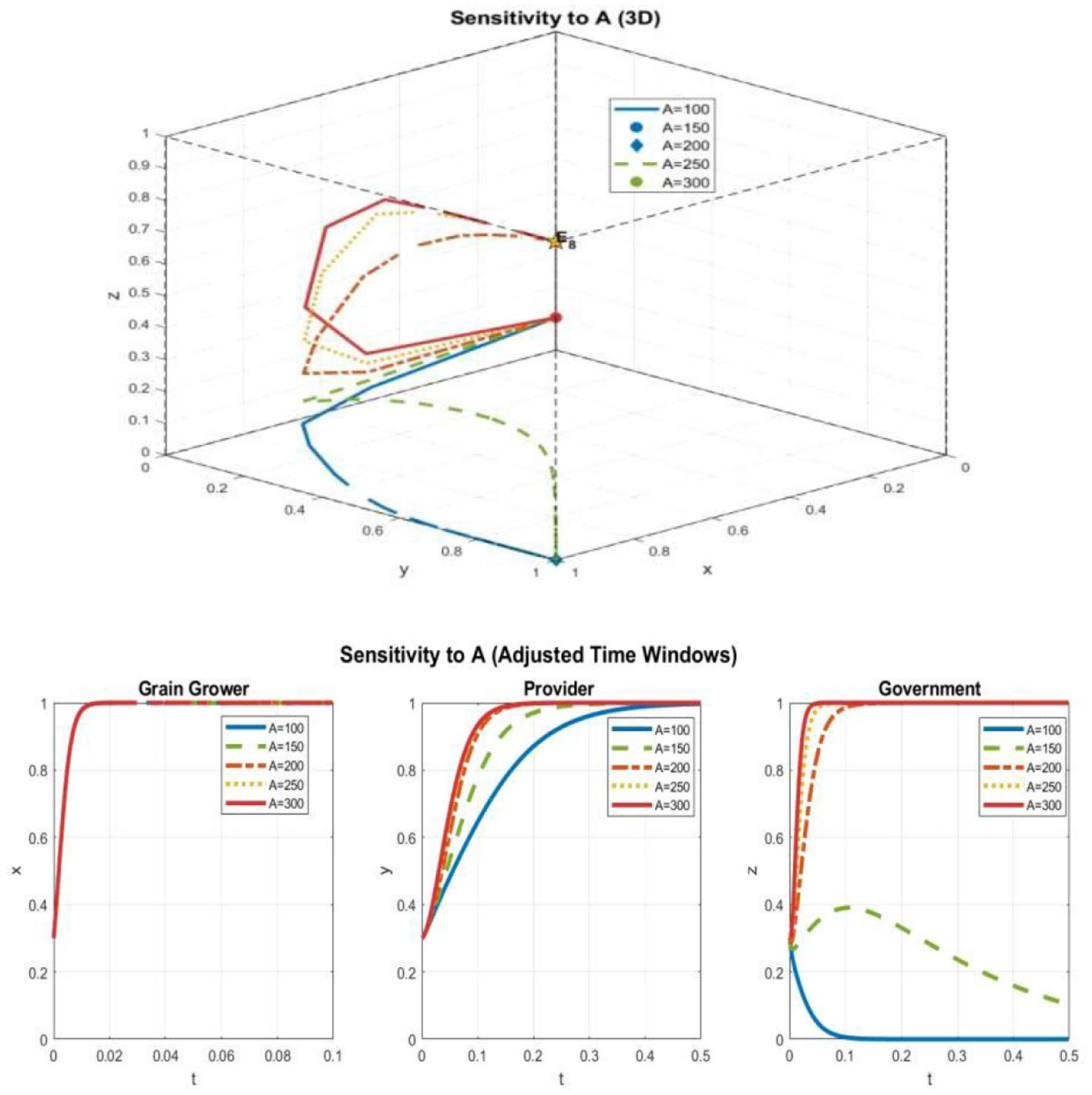
Impact of changes in A on the strategic choices of the three parties.

Central government incentive *A* is a top-level policy instrument used to encourage local governments to actively promote the development of digital agriculture; it directly determines the net payoff of their strong support strategy. Taking the baseline value *A* = 200 yuan per mu, simulations were conducted with *A* set to 100, 150, 250, and 300 yuan per mu. The resulting 3D evolution trajectories and 2D early-stage time series are shown in Figure 6.

As can be seen from the 3D trajectory plot, when *A* ≥ 200, the system ultimately converges to the ideal steady state E8(1, 1, 1), and tripartite strategy coordination is positive. However, when *A* = 100 and 150, the trajectory endpoints deviate significantly from E8, ultimately converging to E5(1, 1, 0). At this equilibrium, grain producers adopt digital technology and service providers offer strong service, but local governments opt for weak support. The root cause is that, in the replicator dynamics equation *F*(*z*), a smaller A reduces the value of *x*[*A*−(*S*_*sa*_−*S*_*wa*_)]−*C*_*p*_−*y*(*S*_*ss*_−*S*_*sw*_), thereby lowering the local government's net payoff. When A is sufficiently small, even if *x* eventually reaches 1, *A*−(*S*_*sa*_−*S*_*wa*_) is still insufficient to cover the additional cost *C*_*p*_+*y*(*S*_*ss*_−*S*_*sw*_) associated with strong support, causing to turn negative in the later stages of evolution. As shown in the 2D trajectory plot, the convergence speed for *A* = 300 is significantly greater than for the other values tested. This indicates that central government incentives must be sufficiently large to cover the net cost of strong local support, so that the government can take the lead in driving the overall process and play an active role throughout.

#### 3.3.5 Joint effects of digital empowerment and producer subsidies

**Figure 7:**
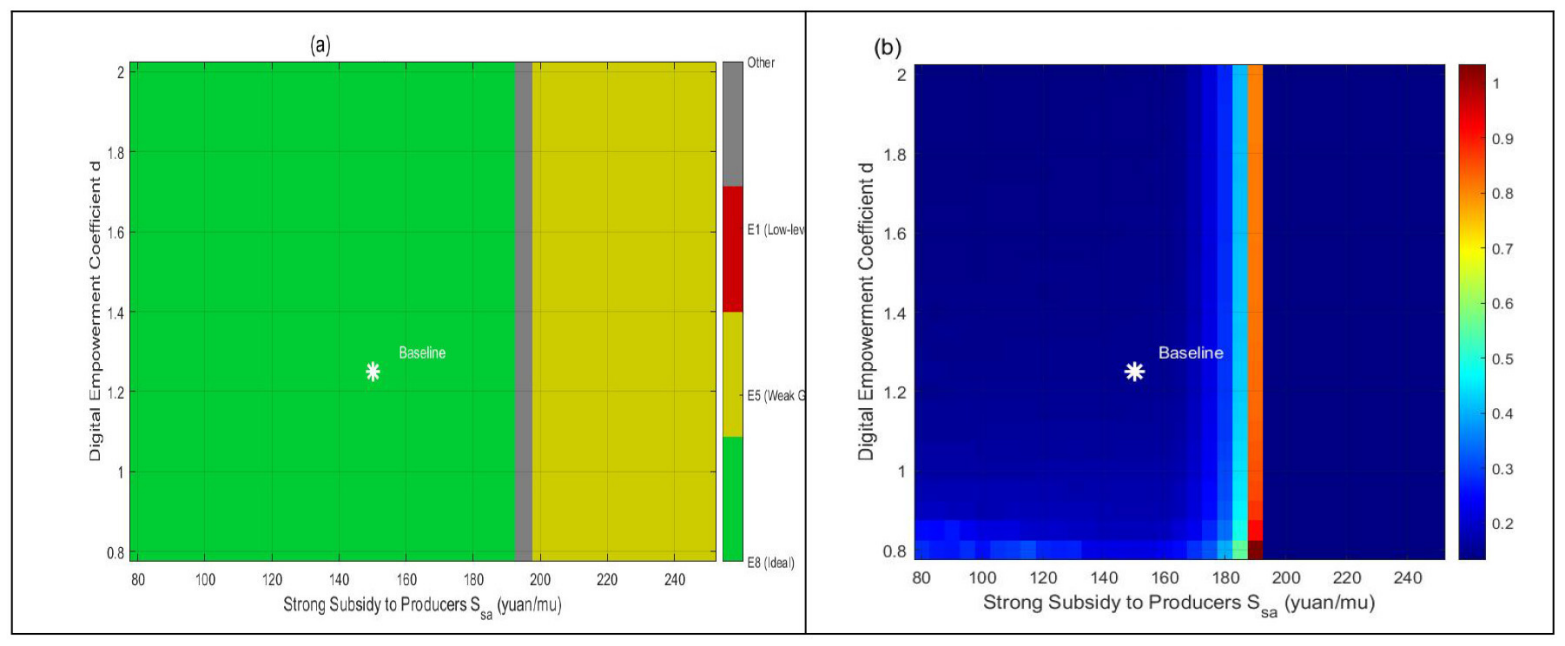
Joint effects of digital empowerment and producer subsidies. **(a)** Steady state classification. **(b)** Convergence time to E8 (t*), white = nonconvergence.

To examine whether technological empowerment can partially substitute for producer subsidies and to uncover their interactive effects, we conducted a two-dimensional parameter scan by jointly varying the digital empowerment coefficient (*d*) and the strong subsidy to grain producers (*S*_*sa*_). For each parameter combination, we simulated the system dynamics from the initial condition (*x*0, *y*0, *z*0) = (0.5, 0.5, 0.5) over a time horizon of *T* = 30. The results are presented in two heat maps: Figure 7a shows the classification of the steady state to which the system converges, and Figure 7b shows the convergence time to E8 (where convergence is defined as *x, y, z* all exceeding 0.99), with white regions indicating non-convergence within 30 periods.

Figure 7 presents the results of a two-dimensional parameter scan jointly varying *d* and *S*_*sa*_. The steady-state classification in Figure 7a reveals three distinct zones. In Zone I (low *d*, low to moderate *S*_*sa*_), the system converges to the low-level equilibrium E1(0, 0, 0), where neither technological appeal nor subsidies is sufficient to trigger adoption. In Zone II (moderate to high *d*, moderate *S*_*sa*_), the system reaches the ideal steady state E8(1, 1, 1), representing tripartite collaboration. In Zone III (moderate to high *d*, excessively high *S*_*sa*_), the system converges to E5(1, 1, 0), where producers adopt, and service providers offer strong services, but local governments withdraw due to fiscal pressure. The negatively sloped boundary between Zones I and II implies that a 0.1 increase in *d* can reduce the minimum *S*_*sa*_ required for E8 by approximately 15–20 yuan per mu, confirming a partial substitutability between technological empowerment and producer subsidies. Yet Zone III indicates that this substitution has a limit: beyond a certain subsidy level, fiscal costs outweigh government benefits, causing support to collapse regardless of technology effectiveness. Figure 7b shows that convergence time to E8 is shortest in the central region of Zone II and increases near the boundaries; notably, when *d* exceeds about 1.45, even a relatively low *S*_*sa*_ can still lead to E8, albeit more slowly. These findings suggest that R&D investment can partially offset fiscal subsidies, but subsidy levels must be carefully calibrated to avoid crowding out government incentives, and policy design should be differentiated according to regional technological conditions.

## 4 Conclusions and policy recommendations

### 4.1 Conclusions

This paper constructs a three-party evolutionary game model involving local governments, digital agriculture service providers and grain producers. It systematically examines the evolutionary mechanisms and steady-state conditions through which digital technology enhances food security, and it analyzes the sensitivity of key parameters via simulation experiments. The main findings are as follows:

(1) The system's evolution exhibits four typical scenarios, each leading to a distinct steady-state outcome. These scenarios are defined by the signs of two net revenue spreads, the net benefit of digital technology adoption for grain producers, and *F*, the net benefit of strong service for providers. Accordingly, the system can be classified into four evolutionary regimes. In Scenario 1 (*E*>0, *F*>0), both the technology and the service market are inherently strong, and the system converges to either E8 or E5. In Scenario 2 (*E*>0, *F* <0), the technology is viable, but service providers' underlying profitability is weak; candidate ESSs include E8, E5, E6, or E4. The final steady state is jointly determined by whether subsidies can offset the service provider's cost disadvantage and by the government's net revenue. In Scenario 3 (*E* <0, *F*>0), service providers have sufficient momentum, but the technology itself lacks appeal. Candidate ESSs are E8, E5, or E3, and the key factor is whether strong service and subsidies can stimulate grain producers' willingness to adopt the technology. In scenario 4 (*E* <0, *F* <0), the system suffers a dual market weakness. Without intervention, it is locked into the low-level equilibrium E1. Only by simultaneously enhancing technological enablement, service profitability, and government incentives can the system transition to E8.

(2) After calibrating the benchmark parameters, we find that China's current agricultural development exhibits the characteristic profile of *E*>0 and *F* <0, and the system possesses an intrinsic dynamic to converge toward the ideal steady state E8. Integrated multi-source data analyses indicate that the yield-and income-enhancing effects of digital technologies on major cereal crops have been widely validated. However, the core business models of field service providers remain relatively fragile. Under these baseline parameters, the initial-path analysis shows that the system converges to the ideal steady state E8 regardless of the proportions of the three parties' initial strategies. This indicates that current policies and market conditions have already generated the fundamental momentum needed to drive tripartite collaboration. The convergence process follows a distinct sequence: grain producers take the lead, local governments follow suit, and service providers lag. This pattern reflects the pressure that market demand exerts on policy-makers and service suppliers when the technical and economic advantages are sufficiently large.

(3) The digital empowerment coefficient, subsidies to primary actors, service providers' operating costs, and central government incentives are the four key levers driving the system's evolution. Sensitivity analysis reveals that the digital empowerment coefficient *d* is the primary driving force; even a slight increase can significantly accelerate grain producers' adoption and global convergence. Generous subsidies for grain producers have an immediate and positive effect on stimulating adoption, but if set too high they crowd out the government's net benefits—hence, an optimal range exists. Service providers' operating cost *C*_*s*_ is a key supply-side bottleneck; reducing it below a critical threshold moves the system from the *F* <0 regime to the ideal *F*>0 regime. The central government incentive A determines the stability of local government strategies; falling below its critical value causes local governments to degenerate into weak support, shifting the system from E8 to E5. These four factors correspond, respectively, to the dimensions of technical effectiveness, institutional incentives, service costs, and government motivation, and together constitute the synergistic focal points driving the system's evolution toward an ideal steady state. Together, they constitute the synergistic levers that drive the system's evolution toward an ideal steady state.

### 4.2 Policy recommendations

Based on the above conclusions, and to effectively encourage grain producers to adopt digital technologies and thereby boost grain production, this paper puts forward the following policy recommendations:

#### 4.2.1 Scenario-based policy design

The four scenarios require fundamentally different policy approaches. When both technology and services are inherently strong, policies should focus on maintaining central incentives above the required threshold and reducing local administrative costs. For regions where technology is effective but service providers face profitability challenges, as in China's major grain-producing regions, policy should prioritize supporting service providers through cost subsidies and expanding adoption scale to unlock additional benefits, while maintaining adequate government incentives. In areas where service supply is strong but technology lacks appeal, the priority is to stimulate producers' willingness to adopt by lowering barriers, enhancing service accessibility, and raising the net benefit of adoption above that of traditional farming. Where both technology and services are weak, systemic intervention is required to simultaneously address deficiencies in both dimensions, necessitating coordinated efforts across all three parties.

#### 4.2.2 Differentiated regional implementation

Given the heterogeneity across grain-producing areas, policy design should be tailored to local conditions. In the Northeast Plain, where service supply is strong but technology adoption lags, resources should be directed toward demonstration projects, training programs, and targeted early-adopter subsidies to raise the net benefit of adoption. In the Yangtze River Basin, where technology is effective but service providers face profitability challenges, policy should focus on cost subsidies, interest subsidies for equipment purchases, and tax incentives to reduce unit service costs. In the mountainous areas of Southwest China, where both technology and services are weak, systemic intervention is necessary, including adapted low-cost digital solutions suitable for hilly terrain, government procurement of services, and coordinated incentives across all three parties.

#### 4.2.3 Calibrating subsidy and incentive levels

Our sensitivity analysis reveals a partial substitutability between technological empowerment and producer subsidies, implying that investments in digital technology research and extension can partially offset the need for fiscal subsidies. However, subsidies beyond a certain level can crowd out government incentives, as excessive fiscal burdens undermine local governments' willingness to maintain strong support. Therefore, subsidy levels should be kept within an optimal range rather than maximized. Similarly, central government incentives must be sufficiently large to cover the net costs of local governments' strong support, preventing them from retreating to weak support. We recommend linking subsidies to performance indicators such as actual yield increases and technology application acreage, shifting from a universal approach to a targeted, performance-based one.

#### 4.2.4 Institutional mechanisms for implementation

To operationalize these recommendations, we propose a cross-departmental joint meeting mechanism, convened regularly and chaired by the agricultural and rural affairs department, to coordinate efforts across agriculture, finance, science and technology, and other relevant sectors. County-level demonstration zones should be established with a clear creation period followed by a consolidation phase for scaling up; funding should come from central fiscal special transfers supplemented by provincial matching funds. Zone performance should be included in the provincial governor's food security responsibility assessment, with key indicators covering adoption rates, service coverage, and yield increases. Zones failing to meet annual targets should receive warnings and be subject to rectification; persistent underperformers should be disqualified and replaced. These institutional arrangements ensure that the policy recommendations are both feasible and accountable.

### 4.3 Limitations of the study and future directions

Although this paper focuses on the role of digital technology in strengthening food security and has conducted a relatively systematic theoretical analysis and simulation by constructing a three-party evolutionary game model, it nonetheless has several limitations that warrant further investigation in future research.

(1) Simplifying model assumptions and refining behavioral depictions. This paper assumes that local governments, digital agriculture service providers, and grain producers are all boundedly rational agents whose strategic choices follow a trial-and-error learning process described by replicator dynamics equations. Although this framework is reasonably consistent with the realities of agricultural technology extension, it does involve a necessary simplification of the three parties' behavioral logic. For example, the objective function for local governments primarily incorporates social benefits and central government incentives, but it does not yet fully capture the complex effects of other political motivations, such as horizontal intergovernmental competition and career incentives for officials. The strategic choices of digital agriculture service providers are simplified into a binary variable, for instance, strong vs. weak service, and thus fail to reflect dynamic decisions about service pricing, technological iteration, and market entry and exit. Moreover, while the technology adoption behavior of grain producers incorporates success probabilities and empowerment coefficients, it does not sufficiently capture micro-level factors such as heterogeneity in producers' risk preferences, social network effects, and information asymmetry. Future research could incorporate behavioral economics tools, including prospect theory and social network analysis, to develop more refined decision-making models for the three parties, thereby enhancing the model's capacity to explain real-world behavior. In addition, the current model assumes that grain producers are homogeneous and make adoption decisions independently. In practice, however, farmers' technology adoption is often influenced by peer behavior and information diffusion through social networks. Future research could relax this assumption by incorporating social network effects or agent-based simulations to capture peer influence and social learning, thereby providing a more realistic representation of the adoption process.

(2) Regional dependence of simulation parameters and the lack of empirical validation. The benchmark parameters used in the simulation analysis are primarily based on macro-statistical data and field surveys conducted in Chongqing and selected major grain-producing regions. Consequently, the parameter values exhibit a degree of regional specificity. Although data from several districts and counties were incorporated into the multi-source fusion calibration process, the generalizability of the model parameters remains to be tested, given substantial differences across China's grain-producing regions in natural endowments, arable land characteristics, farm sizes, and digital infrastructure. Furthermore, the simulation findings lack empirical support from large-scale micro-data. For instance, the existing simulation parameters cannot adequately capture the heterogeneity of the digital empowerment effect across different crops and topographic conditions, nor the non-linear variation in service providers' cost structures relative to operational scale. In addition, the technology accessibility coefficients are treated as exogenous in our model. In contrast, in practice, they evolve dynamically as service provider networks expand and farmers' cumulative learning accumulates over time. Future research could draw on nationwide longitudinal survey data, such as those collected by the Ministry of Agriculture and Rural Affairs, or quasi-natural experiments conducted at smart agriculture demonstration bases, to endogenize these coefficients by incorporating network dynamics and social learning mechanisms into the modeling framework, and to estimate and calibrate key model parameters with greater statistical representativeness, thereby enhancing the external validity of the findings.

Strengthening the link between evolutionary game theory and empirical research. This paper focuses on theoretical modeling and numerical simulation, which fall under the “ex ante” analytical paradigm of evolutionary game theory, and emphasizes the identification of possible evolutionary trajectories and steady-state conditions. However, whether evolutionary game models can effectively predict real-world trajectories of digital agriculture adoption remains to be determined through meaningful engagement with empirical or comparative case studies. For example, econometric models could be constructed using multi-regional panel data to test whether the causal relationships among subsidy intensity, service provider density, and digital technology adoption rates are consistent with the simulation findings. Alternatively, a number of representative regions could be selected for a multi-case comparison, tracing the actual evolutionary trajectories under different combinations of *E* and *F* parameters and verifying the real-world relevance of the four evolutionary scenarios. This closed-loop research design, consisting of three interconnected phases, namely theoretical deduction, simulation modeling, and empirical testing, represents a key direction for advancing evolutionary game theory from theoretical analysis toward policy application.

## Data Availability

The original contributions presented in the study are included in the article/supplementary material, further inquiries can be directed to the corresponding author.
